# Histone lactylation-boosted AURKB facilitates colorectal cancer progression by inhibiting HNRNPM-mediated PSAT1 mRNA degradation

**DOI:** 10.1186/s13046-025-03498-1

**Published:** 2025-08-11

**Authors:** Yuyi Li, Jinjin Peng, Di Wu, Qingxin Xie, Yichao Hou, Linjing Li, Xintian Zhang, Yu Liang, Jing Feng, Jiaqing Chen, Wangshuang Chen, Che Xu, Han Yao, Xiangjun Meng

**Affiliations:** 1 Department of Gastroenterology, Shanghai Ninth People’s Hospital, School of Medicine, Shanghai Jiao Tong University, Shanghai, China; 2 Shanghai Key Laboratory of Gut Microecology and Associated Major Diseases Research, Shanghai, China; 3 Digestive Disease Research and Clinical Translation Center, Shanghai Jiao Tong University, Shanghai, China

**Keywords:** AURKB, CRC, PSAT1, HNRNPM, Histone lactylation

## Abstract

**Background:**

Aurora kinase B (AURKB), a key regulator of mitosis, is frequently upregulated in various malignancies, including colorectal cancer (CRC), and is associated with poor prognosis. However, the limited clinical efficacy of AURKB inhibitors suggests the existence of previously unrecognized oncogenic mechanisms that merit further investigation.

**Methods:**

AURKB was prioritized through bioinformatic analysis, and its elevated expression in CRC was validated via single-cell RNA sequencing (scRNA-seq) and western blot. The transcriptional activation of AURKB was attributed to H3K18 lactylation, as confirmed by chromatin immunoprecipitation (ChIP)-qPCR. RNA sequencing (RNA-seq) and gene set enrichment analysis (GSEA) were conducted to pinpoint the downstream targets of AURKB. The role of the AURKB/phosphoserine aminotransferase 1 (PSAT1) axis in CRC was further studied using both in vitro and in vivo functional experiments. Mass spectrometry, co-immunoprecipitation (Co-IP), proximity ligation assay (PLA), RNA immunoprecipitation (RIP)-qPCR, and mRNA stability assays were employed to investigate the interplay and potential mechanisms involving AURKB, heterogeneous nuclear ribonucleoprotein M (HNRNPM), and PSAT1.

**Results:**

AURKB was identified as an oncogene linked to advanced pathological staging and poor clinical outcomes in CRC. Its transcriptional upregulation was driven by H3K18 lactylation at its promoter. PSAT1 was further identified as a key downstream effector in AURKB-mediated CRC progression. Mechanistically, AURKB bound to HNRNPM and interfered with its interaction with PSAT1 mRNA, thereby suppressing HNRNPM-mediated mRNA degradation and ultimately increasing PSAT1 protein levels.

**Conclusion:**

Our findings uncover a previously unappreciated, kinase-independent function of AURKB in CRC, redefining its therapeutic relevance beyond kinase inhibition. This highlights the need for broader targeting strategies, including PROTAC-mediated degradation of AURKB and pharmacological inhibition of the AURKB/PSAT1 axis, to fully harness its role in CRC treatment.

**Supplementary Information:**

The online version contains supplementary material available at 10.1186/s13046-025-03498-1.

## Introduction

Colorectal cancer (CRC) ranks second in incidence and fourth in mortality among all malignancies in China. Globally, CRC remains a major public health concern, with approximately 1.93 million new cases and 900,000 deaths reported in 2022 [1]. Despite notable progress has been made in CRC treatment, including surgery, chemotherapy, radiotherapy, immunotherapy, and molecular targeted therapy, the prognosis remains poor, with a 5-year overall survival (OS) rate hovers around 14% for patients with metastatic lesions [2]. Therefore, a comprehensive interrogation of the molecular mechanisms driving CRC progression is essential for the development of innovative therapeutic strategies and the improvement of patient prognosis.

Aurora kinase B (AURKB), the catalytic core of the chromosomal passenger complex (CPC), orchestrates key processes during mitosis, including chromosome condensation, kinetochore-centromere attachment, spindle assembly checkpoint, and cytokinesis [3, 4]. Previous evidence has shown that AURKB is often overexpressed in various malignancies, including CRC. Elevated AURKB expression is strongly correlated with tumor proliferation, invasion, metastasis, and drug resistance, making it a robust predictor of poor clinical outcomes [5]. Mechanistically, AURKB promotes cell cycle progression by phosphorylating histone H3 at the Ser10 residue to activate CCND1 and CCNE1 transcription [6, 7]. It also impedes apoptosis by modifying BIM and Survivin [8, 9]. Moreover, AURKB enhances oncogenic signaling by stabilizing MYC through phosphorylation at Ser67 and Ser373 [10, 11], and by accelerating p53 degradation via phosphorylation at Ser183, Thr211, and Ser215 [12].

Despite its oncogenic potential, AURKB-targeted monotherapies have yielded limited efficacy and are often accompanied by substantial adverse effects [13–17]. These clinical challenges highlight the complexity of AURKB’s role in tumorigenesis, potentially involving kinase-independent functions and complex regulatory interactions. A deeper understanding of these mechanisms is imperative to unlock its full therapeutic potential.

In this study, AURKB was identified as a putative oncogene implicated in CRC progression and poor prognosis. We demonstrate that histone H3 lysine 18 lactylation (H3K18la) drives the transcriptional upregulation of AURKB in CRC. Furthermore, Elevated AURKB interacts with heterogeneous nuclear ribonucleoprotein M (HNRNPM) to stabilize PSAT1 mRNA, thereby enhancing serine biosynthesis and facilitating CRC progression. These findings reveal a novel non-canonical function of AURKB in CRC, offering insight into its resistance to conventional kinase-targeted therapies and suggesting new therapeutic avenues.

## Methods and materials

### Data sources and differential expression analysis

The RNA sequencing (RNA-seq) data and corresponding clinical information from the Cancer Genome Atlas (TCGA) project of Colon Adenocarcinoma (COAD) were sourced from the University of California, Santa Cruz Xena browser (http://xena.ucsc.edu/). Additional CRC transcriptomic datasets, GSE17536 and GSE39582, along with associated clinical metadata were retrieved from the Gene Expression Omnibus (GEO) database (https://www.ncbi.nlm.nih.gov/gds). Differentially expressed genes (DEGs) within the TCGA-COAD cohort were identified using the DESeq2 package in the R programming environment (v4.2.1). For GEO datasets (GSE74602, GSE20916, and GSE39582), DEGs between CRC and adjacent normal tissues were obtained using the GEO2R online analysis tool.

### Weighted gene co-expression network analysis

Weighted gene co-expression network analysis (WGCNA) was employed to construct the DEGs co-expression network. Briefly, sample clustering was performed using the average linkage method to detect and exclude outlier samples. An optimal soft-thresholding power (β) was selected to achieve a scale-free topology fit index of 0.9. The resulting adjacency matrix was converted into a topological overlap matrix (TOM), from which a dissimilarity matrix (1-TOM) was derived. This dissimilarity matrix was then used to generate a gene clustering dendrogram, with a minimum module size set to 30. Highly similar dynamic modules were merged using a cut height of 0.25. Pearson correlation analysis was subsequently conducted to assess the associations between gene modules and clinicopathological traits.

### Protein-protein interaction network construction

Protein-protein interaction (PPI) networks were constructed using the STRING database (http://www.string-db.org), which integrates known and predicted interactions. Network visualization and analysis were performed using Cytoscape software (v3.10.2). To identify key regulatory genes, the CytoHubba plugin was employed, ranking nodes based on their degree of connectivity within the network.

### Gene set enrichment analysis

Gene set enrichment analysis (GSEA) was conducted using GSEA software (v4.3.2) to identify biological pathways activated by AURKB in CRC. For this analysis, the curated gene set collection (c2.cp.kegg_legacy.v2024.1.Hs.symbols.gmt) was sourced from the Molecular Signatures Database (http://www.broad.mit.edu/gsea/msigdb/). Gene sets with a *p*-value < 0.05 were deemed statistically significant.

### Functional and pathway enrichment analysis

Gene identifiers were mapped using the org.Hs.eg.db package. Functional enrichment and pathway analysis were performed using the R package clusterProfiler. including Gene Ontology (GO) terms and Kyoto Encyclopedia of Genes and Genomes (KEGG) pathway.

### Single-cell RNA sequencing analysis

Single-cell RNA sequencing (scRNA-seq) data were processed using the Seurat package in R for quality control and downstream analysis. Low-quality cells were excluded based on three criteria: fewer than 200 detected genes, genes expressed in fewer than 10 cells, or mitochondrial gene content exceeding 30% of the total expression. The filtered dataset was normalized, and the top 3000 highly variable genes were selected for dimensionality reduction via principal component analysis (PCA). The first 20 principal components, determined using the elbow method, were used to compute t-distributed stochastic neighbor embedding (t-SNE) for visualization. Clustering was performed using the FindNeighbors and FindClusters functions, with a resolution parameter of 0.3. Cell types were manually annotated by comparing cluster–specific marker genes with reference markers from published studies and the CellMarker database (http://bio-bigdata.hrbmu.edu.cn/CellMarker/). Visualization of target gene expression across clusters was conducted using the FeaturePlot and VlnPlot functions embedded in Seurat.

### Molecular docking

Crystal structures of AURKB (PDB ID: 5K3Y) and HNRNPM (PDB ID:2OT8) were retrieved from the Protein Data Bank (https://www.rcsb.org/*).* Water molecules and native ligands were removed manually using PyMOL software (v3.0.3). Protein-protein docking between AURKB and HNRNPM was conducted using the GRAMM-X online server (http://gramm.compbio.ku.edu/*).* Intermolecular interactions were subsequently analyzed and visualized using PDBePISA (https://www.ebi.ac.uk/pdbe/pisa/) and PyMOL.

### Clinical samples

All patients were pathologically diagnosed with CRC. Tissue specimens used for RNA or protein extraction were snap-frozen and stored at -80 °C, while those for immunohistochemical (IHC) staining were formalin-fixed and paraffin-embedded. None of the patients received preoperative chemotherapy or radiotherapy. Written informed consent was obtained from each participant, and the study was approved by the Ethics Committee of the Ninth People’s Hospital Affiliated with Shanghai Jiao Tong University School of Medicine. Detailed clinical information is provided in Table S1.

### Cell lines

Human embryonic kidney cell line 293T, CRC cell lines HCT116, RKO, LoVo, SW1116, SW480, DLD1, and the normal colonic epithelial cell line NCM460 were obtained from the American Type Culture Collection (ATCC). All cell lines tested negative for mycoplasma contamination. HCT116 and SW1116 were cultured in McCoy’s 5 A medium (Gibco, USA); RKO, SW620, DLD1 and 293T in high-glucose DMEM medium (Gibco, USA); LoVo in Ham’s F-12 K medium (Gibco, USA); SW480 and NCM460 in RPMI-1640 medium (Gibco, USA). All media were supplemented with 10% fetal bovine serum (FBS; Vazyme, China) and 1% penicillin-streptomycin sulfate (Gibco, USA), and cells were maintained in a humidified incubator at 37℃ with 5% CO2.

### Western blot

Western blot was conducted as previously described [18], using β-actin (ACTB) as an internal loading control. Detailed information on the primary antibodies is provided in Table S2.

### Co-immunoprecipitation and mass spectrometry protein identification

Testes or transfected cells were lysed in Pierce IP Lysis Buffer (Thermo Scientific, USA) supplemented with protease inhibitors (Beyotime, China) and incubated on ice for 40 min. Supernatants were incubated with 1 µg of the indicated antibodies at 4 °C under gentle rotation overnight. Protein complexes were pulled down using Protein A/G agarose beads (Thermo Scientific, USA) for 4 h at 4 °C. Immunoprecipitated samples were washed five times with ice-cold PBS containing 0.1% Tween-20, and the bound proteins were eluted by heating in 30 µL of 2×loading buffer at 95 °C for 10 min. Eluates were resolved by SDS-PAGE for western blot analysis. For mass spectrometry (MS) analysis, immunoprecipitated samples bound to beads (without elution) were directly sent to Jingjie PTM BioLab (Hangzhou, China) for downstream processing.

### Cell apoptosis assay

Cell apoptosis was assessed using a double-staining kit with Annexin V/PI or Annexin V/7-AAD (BD Biosciences, USA). Cells were harvested, washed twice with ice-cold PBS, and resuspended in 1×binding buffer at a concentration of 1 × 10^6 cells/mL, according to the manufacturer’s instructions. The cell suspension was then stained with 4 µL Annexin V and 8 µL PI (or 2 µL 7-AAD) and incubated in the dark at room temperature for 15 min. Flow cytometry was performed within 1 h to quantify apoptotic cells.

### RT-qPCR

RT-qPCR was performed as described in previously study [19], using GAPDH as the internal control. Relative gene expression was analyzed by the 2^−ΔΔCt^ method. All primers were synthesized by Sangon Biotech (Shanghai, China) and are listed in Table S3.

### Semi-quantitative RT-PCR

To assess alternative splicing, primers flanking the differentially spliced region were designed using the Primer-BLAST tool (https://www.ncbi.nlm.nih.gov/tools/primer-blast/) and are provided in Table S3. Isoforms containing or excluding the target exon were amplified using 2×Taq Master Mix (Dye Plus; Vazyme, China). PCR products were resolved on a 2% agarose gel in TAE buffer at 150 V for 40 min. Quantification of splicing events was performed by densitometric analysis of gel bands using ImageJ software (v1.53), and percent spliced-in (PSI) values were calculated accordingly.

### Immunohistochemical staining

IHC staining was conducted on formalin-fixed, paraffin-embedded tissue sections to detect AURKB, PSAT1, Ki67,cleaved-caspase3 (c-caspase3), pan lysine lactylation (PanKla) and H3K18la. Sections were deparaffinized in xylene and rehydrated through graded ethanol solutions. Antigen retrieval was performed by heating slides in 0.01 mol/L citrate buffer (PH = 6.0) for 15 min. After cooling, endogenous peroxidase activity was blocked by incubating slides in 3% hydrogen peroxide for 15 min at room temperature. Following PBS washes, sections were blocked with goat serum for 1 h at room temperature and then incubated overnight at 4 °C with primary antibodies (Table S2) diluted in buffer. After another PBS wash, HRP-conjugated secondary antibodies were applied for 1 h at room temperature. Quantification was conducted by imaging three randomly selected fields using a DM2000 LED microscope (Leica, Germany).

### Cellular immunofluorescence assay

CRC cells were seeded at approximately 2 × 10^4 cells per chamber in an 8-well chamber slide (Nalge Nunc International, USA) one day before the assay. After removing the supernatant, cells were washed twice with PBS and fixed with 4% paraformaldehyde for 15 min at room temperature. Non-specific binding was blocked with 1% BSA and 0.2% Triton X-100 for 1 h at room temperature. Primary antibodies (Rabbit anti-AURKB, 1:200; Mouse anti-HNRNPM, 1:60) were added and incubated overnight at 4 °C in a humidified chamber. The following day, cells were exposed to secondary antibodies for 30 min in the dark: Alexa Fluor^®^ 555-labeled Donkey Anti-Rabbit IgG (H + L) (Abcam, USA) and Alexa Fluor^®^ 488-labeled Donkey Anti-Mouse IgG (H + L) (Abcam, USA). Nuclei were counterstained with DAPI and mounted using antifade reagent (Beyotime, China) to preserve fluorescence. Slides were sealed with coverslips and visualized under a DMI8 confocal microscope (Leica, Germany).

### Tissue immunofluorescence assay

Paraffin-embedded tissue sections were first deparaffinized and subjected to antigen retrieval, followed by blocking of endogenous peroxidase activity. Nonspecific binding was inhibited by incubating the sections in 5% BSA in PBS for 20 min at room temperature. Sections were then incubated overnight at 4 °C with rabbit anti-AURKB (1:200), followed by HRP-conjugated Goat Anti-Rabbit IgG (H + L) (Abcam, USA). Signal amplification was achieved using Tyramide-488 (Bry-Try488; Runnerbio, China) for 30 min at room temperature. For the second round of staining, sections underwent microwave-based antigen retrieval, were re-blocking with 5% BSA, and incubated overnight at 4 °C with mouse anti-HNRNPM (1:60). Sequential incubation with HRP-conjugated anti-mouse IgG (H + L) (Abcam, USA) and Tyramide-Cy3 (Bry-TryCY3; Runnerbio, China) was performed at room temperature. Finally, nuclei were counterstained with DAPI and mounted with antifade medium (Beyotime, China). Imaging was conducted using a DMI8 confocal microscope (Leica, Germany).

### Proximity ligation assay

Proximity ligation assay (PLA) was applied to examine AURKB-HNRNPM interactions in formalin-fixed paraffin-embedded sections of CRC and paired normal tissues. Sections were deparaffinized, rehydrated, and subjected to standard antigen retrieval. After blocking for 30 min, sections were incubated overnight at 4 °C with primary antibodies (rabbit anti-AURKB, 1:400; mouse anti-HNRNPM, 1:100). The following day, PLA probes (anti-mouse PLUS, DUO92001; anti-rabbit MINUS, DUO92005) were applied at 37 °C for 1 h to bind the primary antibodies. PLA oligonucleotides were then hybridized and circularized by ligation for 30 min at 37 °C. Amplified signals were visualized as red fluorescent puncta and imaged using a Pannoramic MIDI panoramic scanner (3D HISTECH, Hungary).

### Chromatin immunoprecipitation

The chromatin immunoprecipitation (ChIP) assays were performed using an EZ ChIP Chromatin Immunoprecipitation Kit (Millipore, USA) in accordance with the manufacturer’s instructions. Briefly, cells were cross-linked with 1% formaldehyde and quenched with glycine. The cells were then harvested on ice using ChIP lysis buffer, and chromatin was fragmented by sonication (Sonics, USA) to yield DNA fragments ranging from 100 to 1000 bp. Chromatin lysates were immunoprecipitated overnight at 4 ℃ on a rotator using anti-H3K18la or control IgG antibody, followed by a 1 h incubation with protein A/G agarose beads. Immunoprecipitated DNA was purified with spin columns and analyzed by qPCR. Primers sequences used for ChIP-qPCR are provided in Table S3.

### RNA immunoprecipitation

RNA immunoprecipitation (RIP) was carried out using the Magna RIP RNA-Binding Protein Immunoprecipitation Kit (Geneseed, China) following the manufacturer’s instructions. Briefly, 1 × 10^7 cells were harvested and resuspended in 1 mL buffer A supplemented with 1% protease inhibitor and RNase inhibitor. The cell lysate (450 µL) was incubated overnight at 4 °C with gental rotation, using 4 µg of HNRNPM antibody (Proteintech, China) or control IgG antibody (Abclonal, China), together with protein A/G magnetic beads. Post-incubation, bead complexes were washed five times with wash buffer. The immunoprecipitated RNA was then isolated and quantified by RT-qPCR to measure the relative expression level of PSAT1 mRNA.

### RNA sequencing

Total RNA was isolated from RKO cells treated with shNC (*n* = 3) or shAURKB (*n* = 3) and subjected to RNA-seq. Raw sequencing data were generated using the Illumina Novaseq 6000 high-throughput sequencing platform. Differential gene expression analysis was performed using the DESeq2 package. Functional enrichment of DEGs was assessed using GO term and KEGG pathway analyses.

### Xenograft tumor assay

All animal experiments were approved by the Ethics Committee of the Ninth People’s Hospital Affiliated with Shanghai Jiao Tong University School of Medicine (Approve No. SH9H-2023-A775-1). Male BALB/c nude mice (4 weeks old) were obtained from GemPharmatech (Nanjing, China) and housed in single-sex cages under standard laboratory conditions (21 ± 2 °C, 12 h light/dark cycle). A total of 5 × 10^6 stably transfected HCT116 cells were subcutaneously injected into the right axillary region of each nude mouse. Tumor formation and growth were monitored every three days using calipers, and volume was measured using the formula: Volume = [length×width^2] / 2. After 18 days, the mice were sacrificed, and the tumors were excised, weighed, and fixed in 4% paraformaldehyde at room temperature or stored at -80℃ for further analysis.

### Plasmid and siRNA transfection

Plasmids and small interfering RNAs (siRNAs) were purchased from Jikai Gene (Shanghai, China) and Sangon Biotech (Shanghai, China), respectively. For plasmid transfection, cells were seeded in 6-well plates and cultured to 40% confluency. Plasmid DNA (1 µg) was transfected into cells using FuGene HD transfection reagent (Promega, USA) following the manufacturer’s protocol. For siRNA transfection, cells were seeded in 12-well plates the day prior to the transfection. siRNA (40 pmol) was diluted in Opti-MEM media (Gibco, USA) and mixed with 2 µL transfect-mate reagent (GenePharma, China). The mixture was added to each well when cell confluency reached approximately 50%. The sequences of siRNA are provided in Table S3.

### Lentivirus transfection

Lentiviral particles expressing shAURKB, shHNRNPM (sequence listed in Table S3) or overexpressing PSAT1 were purchased from OBio Technology (Shanghai, China). Cells were seeded at a density of 1 × 10^5 cells per well in 12-well plates and cultured for 16 h. The lentiviral solution was added to the culture medium at the recommended multiplicity of infection (MOI). After 24 h of incubation, the medium was replaced with fresh medium, and the cells were maintained for another 24 h. Stable cell lines were selected using culture medium supplemented with 3 µg/mL puromycin or 30 µg/mL blasticidin.

### mRNA stability assay

To assess mRNA stability, HCT116 and RKO cells were transfected with either HNRNPM or siHNRNPM#2 for 48 h. Subsequently, cells were treated with actinomycin D (ACTD, 5 µg/ml; MedChemExpress, USA) to inhibit transcription. Samples were collected at 0 h, 4 h, and 8 h post-treatment, and total RNA was extracted for cDNA synthesis and qPCR analysis. The remaining RNA levels at each time point were normalized to the 0 h baseline.

### Proliferation assay

Cell proliferation was assessed using CCK-8 and colony formation assays. For the CCK-8 assay, transfected CRC cells were seeded in 96-well plates at a density of 3 000 cells per well and cultured at 37℃ at 0 h, 24 h, 48 h, and 72 h post-seeding. 10 µL of CCK-8 reagent (Beoytime, China) mixed with 90 µL serum-free medium was added into each well. After incubation at 37℃ for 1 h, absorbance at 450 nm was measured to determine cell viability. For the colony formation assay, 1 000 cells were seeded into 6-well plates and cultured for 8 to 12 days. Colonies were fixed with 4% paraformaldehyde and stained with 0.1% crystal violet. After washing with PBS and air-drying, each well was photographed, and the colonies (> 50 cells) were counted using ImageJ software (v1.53).

### Serine detection assay

Intracellular serine levels were detected using a Human Serine ELISA kit (COIBO BIO, China) following the manufacturer’s instructions. After 12 h of starvation culture, cells were collected, resuspended in PBS, and subjected to freeze-thaw cycles to release intracellular contents. The resulting lysates were centrifuged at 2000 rpm for 20 min, and the supernatant was collected. ELISA was performed by sequential addition of reagents, incubation, washing, color development, and reaction termination, as per protocol. Absorbance was measured at 450 nm using a TECAN spectrophotometer (Switzerland) within 15 min of the final reaction step.

### Statistical analysis

All quantitative data are presented as mean ± standard deviation (SD) from at least three independent experiments. Comparisons between the two groups were performed using a two-tailed unpaired Student’s *t*-test. For comparisons involving three or more groups, one-way ANOVA followed by Dunnett’s multiple comparison test was applied. Overall survival (OS) was evaluated using the Kaplan-Meier method, and survival curves were compared using the log-rank test. Association between AURKB expression and clinicopathological or molecular features were analyzed via chi-square test. The Spearman’s correlation test was utilized to evaluate the relationship between continuous variables. All statistical analyses were conducted using GraphPad Prism (v9.4.1) and R (v4.2.1). Statistical significance was defined as follows: ns, *P* > 0.05, *P < 0.05, **P < 0.01, and ***P < 0.001.

## Results

### AURKB is a potential driver gene in the progression and poor prognosis of CRC

**Fig. 1:**
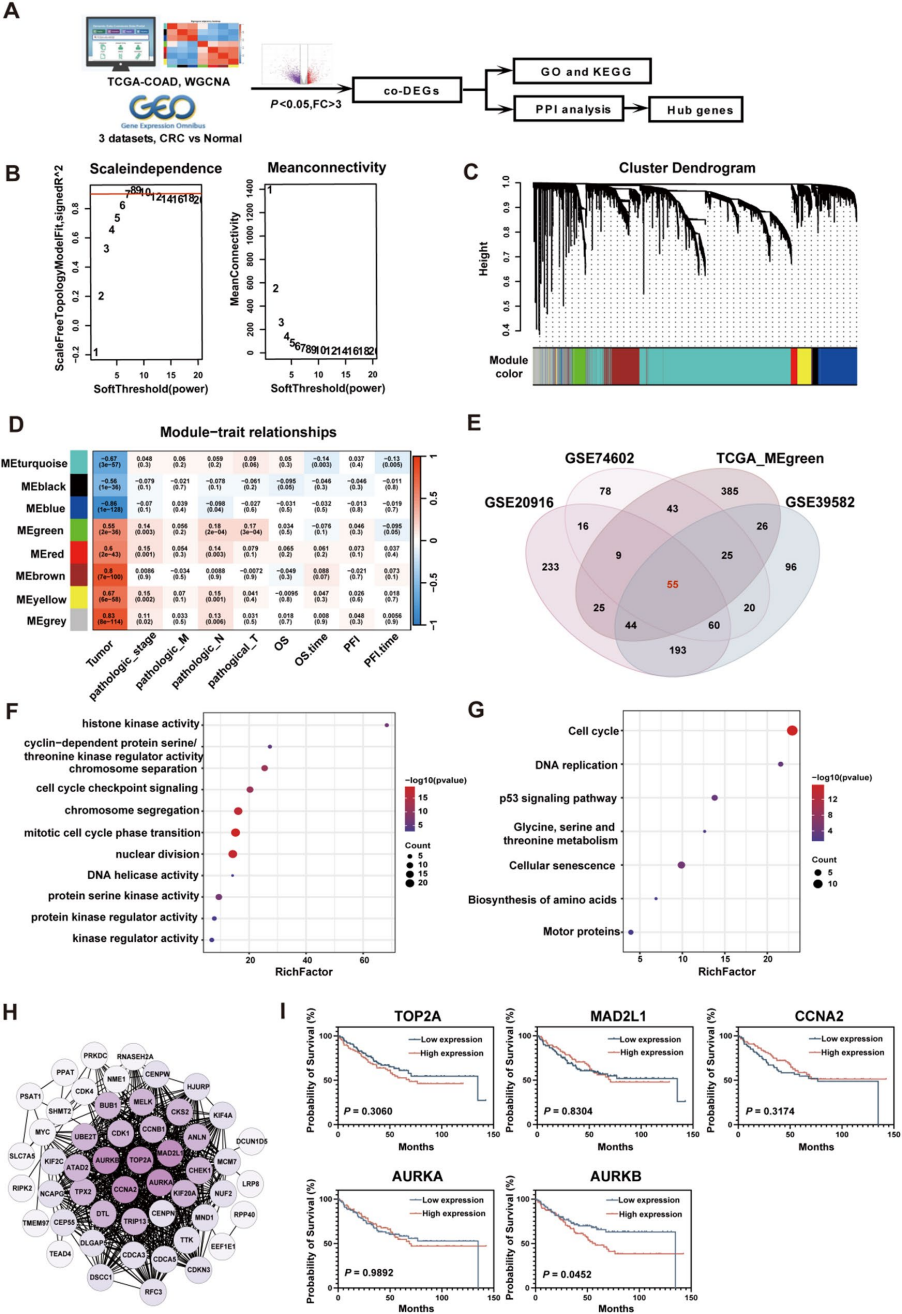
AURKB is a potential driver gene associated with progression and poor prognosis in CRC. (**A**) Workflow of hub gene identification in CRC. A weighted gene co-expression network was constructed using DEGs derived from the TCGA_COAD cohort (nTumor = 383, nNormal = 51). Modules significantly correlated with clinicopathological features of CRC were identified, and the corresponding gene sets were extracted. The gene sets were then intersected with consistently upregulated genes across multiple GEO datasets. GO annotation, KEGG pathway enrichment analysis, and PPI network analysis were subsequently performed to elucidate the molecular mechanisms of these overlapping genes and to identify candidate hub genes. (**B**) Scale independence and mean connectivity analysis. A soft-thresholding power of 7 was chosen to achieve a scale-free fit index of 0.9. (**C**) Clustering dendrogram of 4788 DEGs based on the dissimilarity measure 1-TOM. Each branch represents a gene, with different colors indicating distinct co-expression modules. (**D**) A heatmap displaying the correlation between module eigengenes (MEs) and clinicopathological traits of CRC patients. Each cell shows the correlation coefficient and corresponding *p*-value. (**E**) Venn diagram illustrating the overlap between genes in the WGCNA green module and those highly expressed in CRC tissues across other GEO datasets. (**F-G**) Bubble plots presenting the GO functional annotation (**F**) and KEGG pathway enrichment (**G**) results for the intersecting genes. (**H**) PPI network of the overlapping genes constructed using the STRING database. Each node represents a protein encoded by a distinct gene, with color intensity indicating the degree of protein–protein interaction. Proteins with higher connectivity are positioned centrally within the network. (**I**) Kaplan–Meier survival analysis of the identified hub genes using data from the GSE17536 cohort (*n* = 177) to evaluate their prognostic relevance in CRC. *P*-values were determined using the log-rank test

The workflow for identifying potential driver genes involved in CRC carcinogenesis and progression is illustrated in Fig. 1A. RNA transcriptome data from 383 tumor tissues and 51 normal tissues were extracted from the TCGA-COAD dataset. A total of 4788 DEGs were identified, comprising 1572 upregulated and 3216 downregulated genes. WGCNA was performed on these DEGs, incorporating multiple clinical parameters, including disease status (Tumor/Normal), pathological stage, T/N/M staging, overall survival (OS) status and time, and progression-free survival (PFS) status and time. A soft threshold power of 7 was selected to generate a scale-free network, achieving a high scale independence degree near 0.9 and a low mean connectivity tending to 0 (Fig. 1B). This process yielded eight different co-expressed modules: turquoise, blue, red, brown, yellow, gray, green, and black, as shown in the dendrogram (Fig. 1C). The green module, comprising 612 genes, exhibited a significant correlation with disease status, pathological stage, tumor size, and lymph node metastasis (Fig. 1D). To further refine candidate genes, we conducted an integrative analysis using GE2OR to identify significantly upregulated genes across three GEO datasets. These genes were intersected with those in the green module (MEgreen), leading to a core gene signature associated with CRC progression (Fig. 1E). GO enrichment revealed involvement in cell cycle checkpoint signaling, protein serine kinase activity, and histone kinase activity (Fig. 1F). KEGG analysis indicated enrichment in pathways related to cell cycle regulation, DNA replication, amino acid metabolism, and cellular senescence (Fig. 1G). A protein–protein interaction (PPI) network was constructed, and hub genes were identified using the cytoHubba plugin based on connectivity degree. Five hub genes emerged: AURKA, AURKB, TOP2A, CCNA2, and MAD2L1 (Fig. 1H). Prognostic evaluation revealed that among these, only elevated AURKB expression was significantly associated with poorer OS in CRC patients (Fig. 1I).

To validate the clinical relevance of AURKB, we analyzed its expression in an independent GEO cohort (GSE39582), which included 585 CRC samples. High AURKB expression correlated with more advanced N stage (*P* = 0.014) and TNM stage (*P* = 0.017). In line with its known role in mitotic regulation, a potential trend toward association with chromosomal instability (CIN) was also observed, though this did not reach statistical significance (*P* = 0.087) (Table S4). Overall, these findings position AURKB as a compelling candidate driver gene in CRC, robustly linked to tumor progression and unfavorable prognosis.

### AURKB knockdown inhibits CRC proliferation and induces apoptosis

**Fig. 2:**
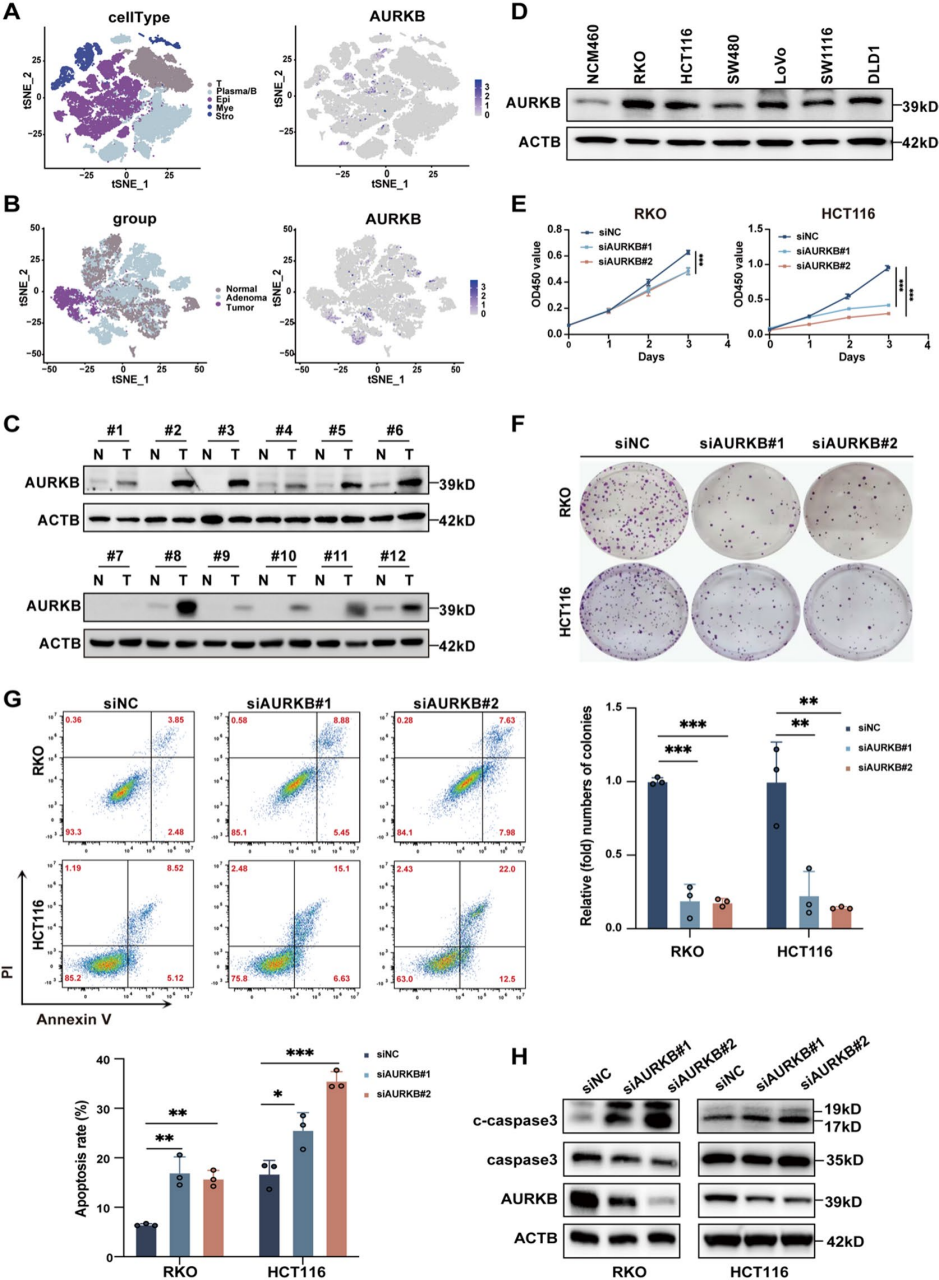
Knockdown of AURKB inhibits CRC cell proliferation and promotes apoptosis. (**A**) t-SNE dimensionality reduction plot of scRNA-seq data from our center, colored by five major cell lineages: T cells, plasma/B cells, epithelial cells, myeloid cells, and stromal cells. AURKB expression is predominantly enriched in epithelial cells. (**B**) t-SNE plot of scRNA-seq data from different groups, showing that cells with high AURKB expression are mainly derived from CRC or colorectal adenoma tissues. (**C**) AURKB expression in CRC and paired normal tissues (*n* = 12 pairs; N: normal, T: CRC). (**D**) AURKB expression in various CRC cell lines and normal intestinal epithelial cells. (**E-F**) CCK-8 and colony formation assays were used to evaluate the effect of AURKB knockdown on the proliferation ability of RKO and HCT116 cells. (**G**) Flow cytometry analysis of apoptosis rates in RKO and HCT116 cells following AURKB knockdown. (**H**) Western blot analysis of AURKB, caspase3, and c-caspase3 expression levels in RKO and HCT116 cells after AURKB knockdown. Data are presented as mean ± SD of three independent experiments in E-G. Statistical analysis was performed by one-way ANOVA followed by Dunnett’s multiple comparisons test (*P < 0.05, **P < 0.01, and ***P < 0.001)

To explore the roles of AURKB in CRC, we first examined its cellular distribution and expression profile. ScRNA-seq analysis revealed that AURKB was predominantly expressed in the epithelial cells, particularly within adenoma and tumor regions (Fig. 2A and B). Similarly, AURKB was upregulated in CRC tissues compared to paired normal tissues (Fig. 2C), and CRC cell lines showed higher levels of AURKB than normal colonic epithelial cells (Fig. 2D). Next, we validated the biological functions of AURKB by introducing two distinct siRNAs into HCT116 and RKO cells. The effects of AURKB knockdown on cell proliferation and apoptosis were assessed through CCK-8, colony formation, and flow cytometry assays. AURKB knockdown notably suppressed cell proliferation and clonogenic ability in both CRC cell lines (Fig. 2E and F). Moreover, flow cytometry revealed a marked increase in apoptotic cell rates after AURKB knockdown (Fig. 2G), corroborated by western blot analysis showing elevated levels of c-caspase 3 (Fig. 2H). Taken together, these results demonstrate that AURKB knockdown inhibits proliferation and induces apoptosis in CRC.

### H3K18la promotes the transcription of AURKB

**Fig. 3:**
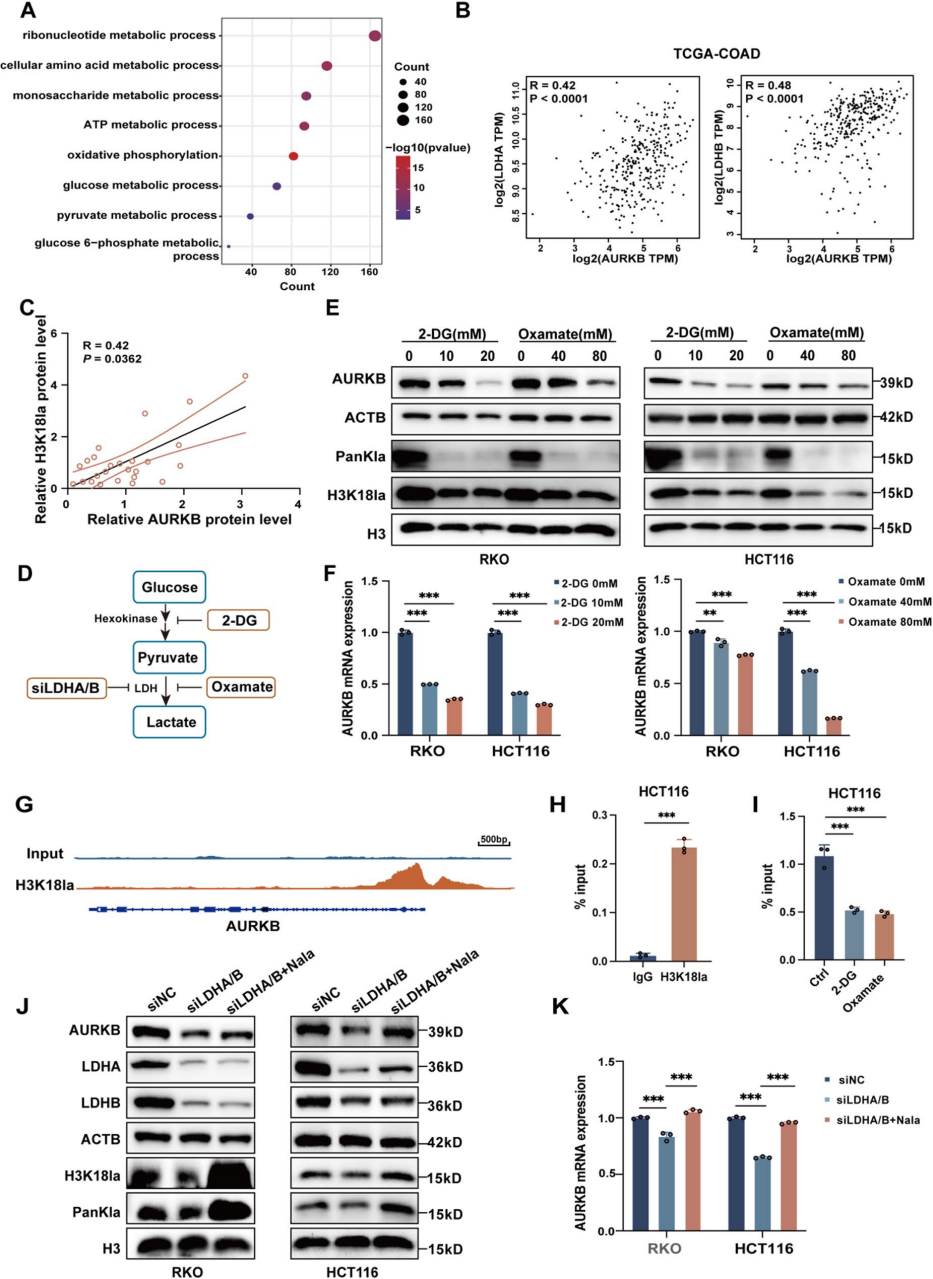
H3K18la promotes AURKB transcription in CRC. (**A**) Genes significantly positively correlated with AURKB expression (Spearman *R* > 0.2 and FDR < 0.05) were extracted from the R2 database. GO analysis revealed significantly enriched terms. (**B**) Correlation analyses between AURKB expression and LDHA or LDHB expression were conducted using GEPIA2 platform (http://gepia2.cancer-pku.cn/), with correlation strength represented by Spearman’s coefficient (*n* = 316). (**C**) Western blot analysis of AURKB and H3K18la protein levels in CRC tissue specimens collected from our center (*n* = 25). Relative expression levels were normalized to ACTB as a loading control. The association between H3K18la and AURKB expression was assessed using Spearman’s correlation analysis. (**D**) Schematic overview of glycolysis, highlighting the experimental interventions used to block the lactate biosynthesis and subsequent histone lactylation in the experimental system. (**E**) Western blot analysis showing the expression of AURKB, PanKla and H3K18la following 48 h treatment with glycolytic inhibitors (2-DG or Oxamate) in RKO and HCT116 cells. (**F**) RT-qPCR quantification of AURKB mRNA levels in RKO and HCT116 cells treated with glycolytic inhibitors (2-DG or Oxamate) for 48 h. (**G**) Representative Integrative Genomics Viewer (IGV) tracks showing H3K18la enrichment at the AURKB promoter region, based on publicly available ChIP-seq data in H1299 cells (GSE207814). (**H-I**) ChIP-qPCR analysis to determine the binding status of H3K18la to the AURKB promoter region in HCT116 cells (**H**). The binding affinity was further evaluated in HCT116 cells treated with 20 mM 2-DG or 80 mM Oxamate (**I**). (**J-K**) RKO and HCT116 cells were subjected to 48 h knockdown of LDHA and LDHB (with or without 25 mM Nala supplementation). Western blot analysis was conducted to evaluate protein levels of AURKB, PanKla, and H3K18la (J), and AURKB mRNA expression was measured using RT-qPCR (**K**). Data are presented as mean ± SD of three independent experiments in F, H, I and K. Statistical analysis was performed by two-sided Spearman’s correlation test in B and C, by two-tailed unpaired Student’s *t*-test in H, or by one-way ANOVA followed by Dunnett’s multiple comparisons test in F, I and K (*P < 0.05, **P < 0.01, and ***P < 0.001)

To interpret the molecular basis for enhanced AURKB expression, we retrieved genes exhibiting expression patterns parallel to AURKB from the R2 database (https://hgserver1.amc.nl/cgi-bin/r2/) (*R* > 0.2, FDR < 0.05). GO analysis revealed that these AURKB-associated genes were significantly enriched in several metabolic processes, including amino acid metabolism, glucose metabolism, and pyruvate metabolism (Fig. 3A). This suggests that the increased AURKB mRNA expression may arise from aberrant metabolic reprogramming. To delineate potential mechanistic connections, the relationship between AURKB and key metabolic enzymes was analyzed, uncovering a significant positive correlation with lactate metabolism enzymes LDHA and LDHB (Fig. 3B). Importantly, histone lactylation, a recently characterized epigenetic modification induced by lactate accumulation, has been implicated in activating oncogene transcription [20, 21]. Consistent with previous reports [20], we detected elevated histone lactylation levels in CRC tissues (Fig. S1A-C), prompting the hypothesis that AURKB overexpression in CRC may be mediated by histone lactylation.

We therefore evaluated the relative expression levels of AURKB, PanKla and H3K18la in CRC specimens collected from our research center. No appreciable correlation was observed between PanKla and AURKB (Spearman *R* = 0.24, *P* = 0.2479; Fig. S1D), whereas H3K18la exhibited a significant positive correlation with AURKB (Spearman *R* = 0.42, *P* = 0.0362; Fig. 3C). Glycolytic inhibitors (2-DG or Oxamate) were subsequently used to examine the effect of decreased lactylation levels on AURKB expression (Fig. 3D). Both inhibitors reduced PanKla and H3K18la levels in CRC cells, concomitant with diminished AURKB mRNA and protein expression (Fig. 3E and F). Conversely, supplementation with sodium lactate (Nala) markedly enhanced AURKB expression (Fig. S1E and S1F). ChIP-seq analysis of publicly available data (GSE207814) identified prominent H3K18la binding peaks at the AURKB promoter in H1299 cells (Fig. 3G), prompting further validation in CRC. ChIP-qPCR in HCT116 cells demonstrated specific H3K18a enrichment at the AURKB promoter, which was profoundly attenuated upon treatment with glycolytic inhibitors (Fig. 3H and I).

To rule out potential off-target effects of pharmacological inhibitors, siRNA-mediated knockdown of LDHA and LDHB was performed to reduce lactate production and cellular lactylation levels (Fig. 3D). Dual silencing (siLDHA/B) effectively reduced PanKla and H3K18la, along with a marked suppression of AURKB expression. Moreover, this repression was partially reversed by Nala supplementation (Fig. 3J and K). Taken together, these findings establish H3K18la as a novel epigenetic regulator that directly enhances AURKB transcription in CRC.

### PSAT1 is a downstream target of AURKB in CRC

**Fig. 4:**
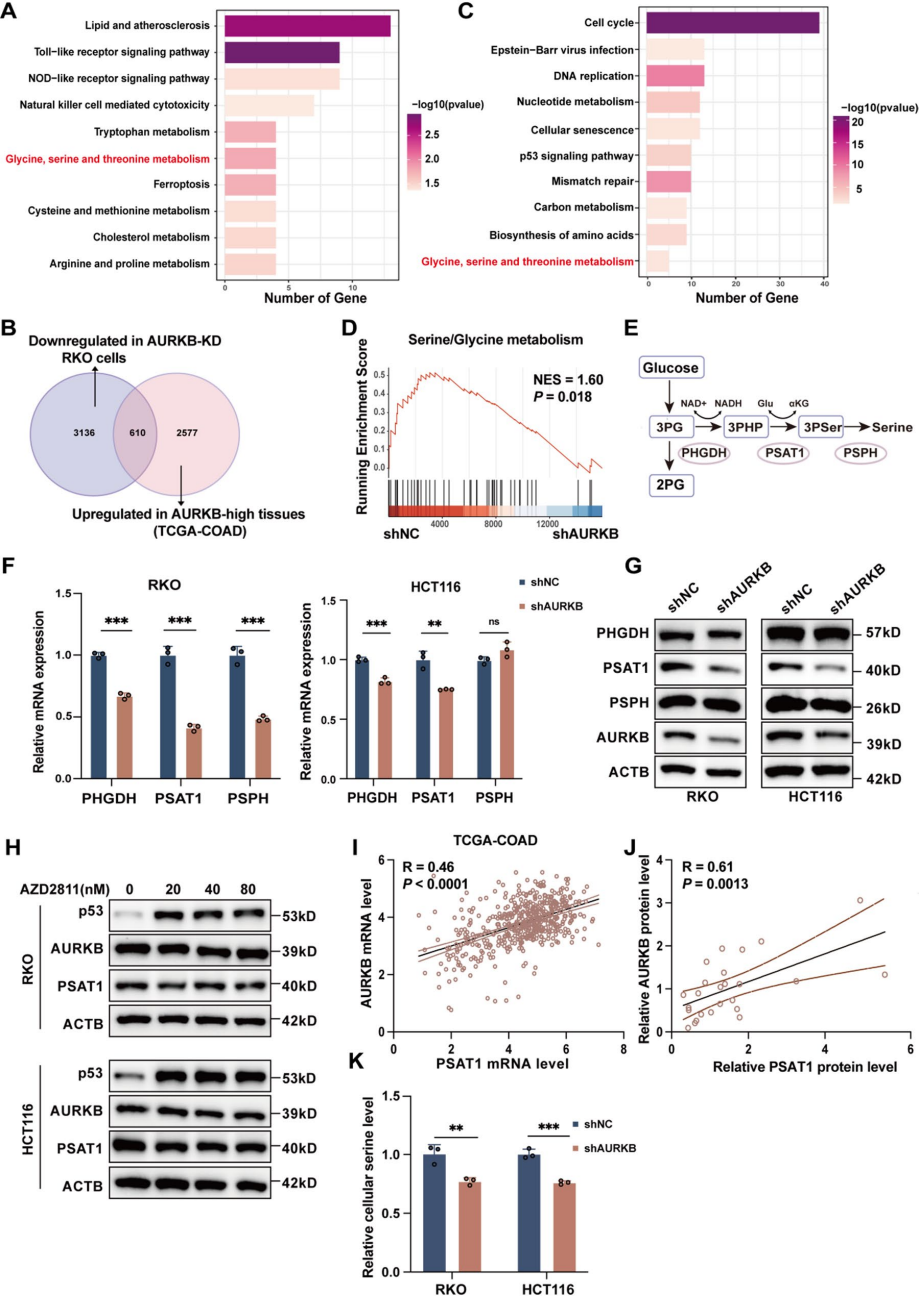
PSAT1 is a potential downstream target of AURKB. (**A**) KEGG enrichment analysis of significantly DEGs in AURKB-knockdown cells compared to control cells (|log2FC| >1 and FDR < 0.05). (**B**) Differential expression analysis of the TCGA-COAD dataset, comparing samples with high versus low AURKB mRNA expression (top 25% and bottom 25%, *n* = 128 per group). A Venn diagram illustrates genes upregulated in AURKB high-expression tissues and downregulated in AURKB-knockdown RKO cells. (**C**) KEGG enrichment analysis of the co-regulated gene set. (**D**) GSEA plot illustrating the enrichment pattern of the serine/glycine metabolic processes. (**E**) Schematic diagram of the *de novo* serine biosynthesis pathway. (**F-G**) RT-qPCR (**F**) and western blot (**G**) analyses were performed to evaluate PHGDH, PSAT1, and PSPH expression in RKO and HCT116 cells following AURKB knockdown. (**H**) RKO and HCT116 cells were treated with varying concentrations of AZD2811 for 48 h, and PSAT1 expression was assessed by western blot, using p53 as a positive control. (**I**) Correlation analysis of AURKB and PSAT1 mRNA expression in the TCGA-COAD cohort (*n* = 512). (**J**) Correlation analysis of AURKB and PSAT1 protein expression in CRC specimens from our cohort (*n* = 25). (**K**) Intracellular serine levels were measured in AURKB stable knockdown RKO and HCT116 cells following 12 h of serum starvation. Data are presented as mean ± SD of three independent experiments in F and K. Statistical analysis was performed by a two-sided Spearman’s correlation test in I and J, or by two-tailed unpaired Student’s *t*-test in F and K (*P < 0.05, **P < 0.01, and ***P < 0.001)

RNA sequencing was conducted on AURKB knockdown and control RKO cells to characterize the downstream biological processes influenced by AURKB in CRC. A total of 247 upregulated genes and 424 downregulated genes were identified based on|log2FC| >1 and FDR < 0.05. KEGG pathway enrichment highlighted significant involvement of these DEGs in immune response, inflammation, and metabolism (Fig. 4A). To extend these findings in patient datasets, CRC samples from the TCGA-COAD cohort were stratified by AUKRB expression into high (top 25%) and low (bottom 25%) groups. This comparison identified 3187 genes significantly upregulated in AURKB-high tumors (FC > 1, FDR < 0.05). A Venn diagram analysis revealed 610 overlapping genes that were consistently downregulated in AURKB-knockdown cells and upregulated in AURKB-high tissues (Fig. 4B). KEGG enrichment of these genes pointed to cell cycle regulation, cellular senescence, DNA replication, and notably, glycine, serine and threonine metabolism (Fig. 4C). GSEA confirmed downregulation of serine and glycine metabolism pathways in AURKB-depleted cells (Fig. 4D). Among these processes, the most prominently dysregulated genes were primarily involved in serine biosynthesis, including PHGDH, PSAT1, and PSPH (Fig. 4E and Table S5). Experimental validation revealed that only PSAT1 was markedly reduced at both the mRNA and protein levels upon AURKB knockdown (Fig. 4F and G). Interestingly, inhibition of AURKB kinase activity with AZD2811 did not alter PSAT1 levels (Fig. S3A and Fig. 4H). Furthermore, expression of a kinase-dead AURKB mutant (K106R) [22] (Fig. S3B) effectively restored both PSAT1 expression (Fig. S3C and S3D) and cell proliferation (Fig. S3E and S3F) suppressed by AURKB depletion, indicating that AURKB regulates PSAT1 through a kinase-independent mechanism.

Clinically, PSAT1 was significantly upregulated in CRC tissues compared to normal tissues (Fig. S2A-C), and elevated expression was correlated with poorer OS (*P* = 0.0343; Fig. S2D). Functional assays demonstrated that PSAT1 overexpression promoted proliferation and inhibited apoptosis in CRC cells (Fig. S2E and S2G). Integrated clinical analyses uncovered strong positive correlations between AURKB and PSAT1 expression in CRC tissues from the TCGA-COAD cohort (Spearman *R* = 0.46, *P* < 0.0001; Fig. 4I) and our clinical center (Spearman *R* = 0.61, *P* = 0.0013; Fig. 4J). To probe the functional consequence of this regulatory axis, intracellular serine levels were measured in stable AURKB-knockdown and control cell lines. Knockdown of AURKB significantly reduced intracellular serine levels (Fig. 4K). Together, these findings underscore the pivotal role of AURKB in modulating PSAT1 expression and downstream serine biosynthesis in CRC.

### PSAT1 mediates AURKB-driven CRC progression

**Fig. 5:**
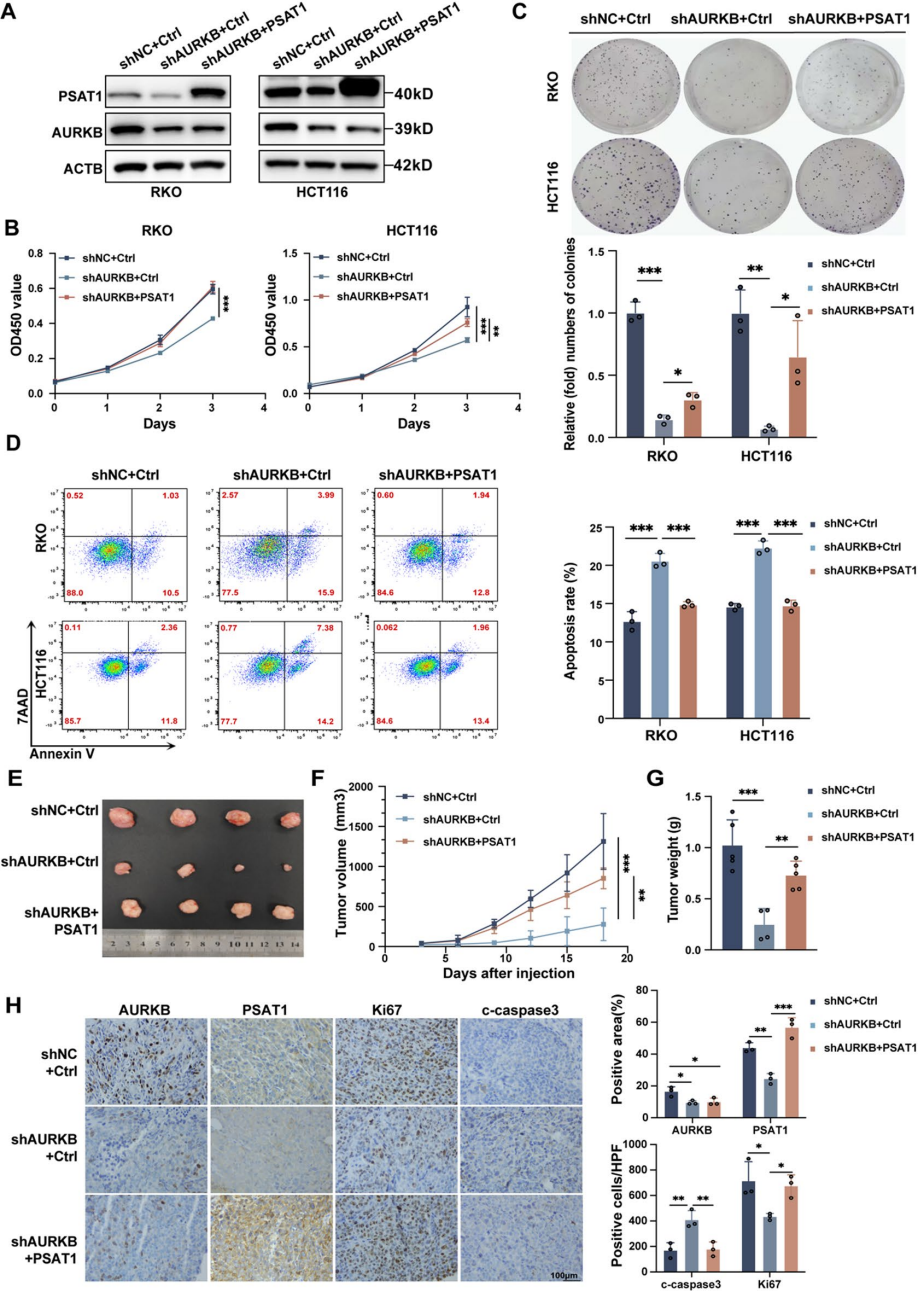
AURKB promotes CRC progression by activating PSAT1 in vitro and in vivo. (**A**) Western blot analysis of AURKB and PSAT1 protein levels in the control group (shNC + Ctrl), knockdown group (shAURKB + Ctrl), and rescue group (shAURKB + PSAT1). (**B-C**) Proliferation ability was assessed using CCK-8 (**B**) and colony formation assays (**C**) across the three groups. (**D**) Apoptosis rates were assessed by flow cytometry, with statistical comparisons among the three groups. (**E-G**) The indicated HCT116 cells were subcutaneously injected into nude mice (*n* = 4–5 per group). Representative tumor images (**E**), tumor growth curves (**F**), and tumor weights (**G**) were recorded and analyzed. (**H**) Representative IHC staining (scale bar = 100 μm) and quantification of AURKB, PSAT1, c-caspase3, and Ki67 expression in xenograft tumors (*n* = 3). Data are expressed as mean ± SD. Statistical analysis was performed by one-way ANOVA followed by Dunnett’s multiple comparisons test in B, C, D, F, G, and H (*P < 0.05, **P < 0.01, and ***P < 0.001)

To elucidate the functional role of PSAT1 in AURKB-mediated CRC progression, PSAT1 expression was restored in AURKB-depleted CRC cells. Ectopic expression of PSAT1 effectively reversed its downregulation caused by AURKB knockdown, while leaving AURKB expression unchanged (Fig. 5A). Reintroduction of PSAT1 also rescued the proliferation defects and apoptosis elevation induced by AURKB depletion, as evidenced by CCK-8, colony formation, and flow cytometry assays (Fig. 5B-D). In vivo validation using xenograft models further confirmed that AURKB knockdown suppressed tumor growth, and this effect was significantly reversed by PSAT1 overexpression (Fig. 5E-G). IHC analysis of tumor tissues revealed reduced PSAT1 and Ki67 expression and elevated c-caspase 3 levels following AURKB knockdown. These molecular changes were restored in the PSAT1 overexpressing group (Fig. 5H). Altogether, these findings highlight that PSAT1 overexpression effectively mitigates the anti-proliferative and pro-apoptotic effects of AURKB depletion, establishing PSAT1 as a key mediator of AURKB-driven CRC progression.

### AURKB interacts with HNRNPM

**Fig. 6:**
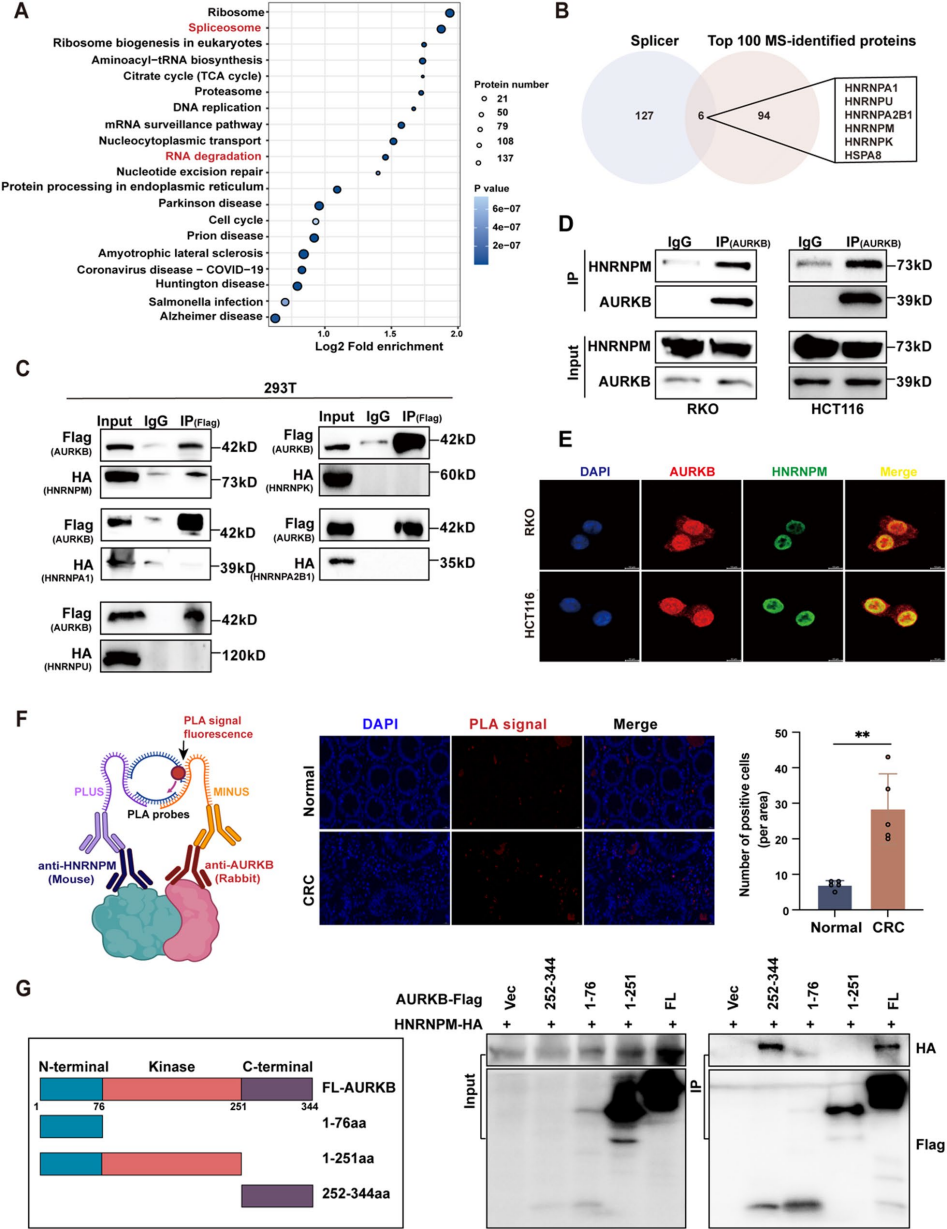
AURKB interacts with HNRNPM. (**A**) KEGG pathway enrichment analysis of AURKB-interacting proteins identified by IP-MS in 293T cells. (**B**) Venn diagram showing the overlap between the top 100 AURKB-interacting proteins and annotated splicing factors. (**C**) 293T cells were co-transfected with HA-tagged HNRNPs and Flag-tagged AURKB plasmids. At 48 h post-transfection, the cells were lysed and subjected to immunoprecipitation using an anti-Flag antibody or control rabbit IgG, followed by western blot with the indicated antibodies. (**D**) Immunoprecipitation with an anti-AURKB antibody was performed, followed by western blot detection of AURKB and HNRNPM in HCT116 and RKO cells. (**E**) Representative fluorescence images showing the co-localization of AURKB (red) and HNRNPM (green) in RKO and HCT116 cells (scale bar = 20 μm), with nuclei counterstained with DAPI (blue). (**F**) PLA assay. Left: Schematic diagram of the principles of PLA (created with BioRender). Middle: Representative images showing the proximity between AURKB and HNRNPM in CRC and paired normal tissues (scale bar = 20 μm). Right: Quantification of PLA signals per area (*n* = 5 randomly selected fields). Data are shown as mean ± SD. Statistical analysis was performed by Student’s *t*-test (*P < 0.05, **P < 0.01, and ***P < 0.001). (**G**) Identification of the essential domain of AURKB for its interaction with HNRNPM. Left: Schematic diagram of truncations of AURKB. Right: Flag-tagged AURKB truncations were co-transfected with HA-tagged HNRNPM into 293T cells. Protein interaction was evaluated by Co-IP assay

To clarify how AURKB regulates PSAT1 expression, we used immunoprecipitation combined with mass spectrometry (IP-MS) to identify proteins that interact with AURKB. GO Biological Process (GO-BP) analysis indicated that these interacting proteins were primarily enriched in RNA splicing and mRNA processing pathways (Fig. S4A). Corroborating these results, KEGG pathway analysis revealed significant enrichment in spliceosome and RNA degradation (Fig. 6A). To refine the analysis, we curated a gene set of splicing factors involved in RNA processing or splicing as defined previously [23], and observed a minor overlap with the top 100 proteins detected by IP-MS: HNRNPA1, HNRNPU, HNRNPA2B1, HNRNPM, HNRNPK, and HSPA8 (Fig. 6B). Given the well-established roles of heterogeneous nuclear ribonucleoproteins (HNRNPs) in RNA processing, HNRNP family members were prioritized for further investigation. Exogenous Co-IP assays confirmed an interaction between AURKB and HNRNPM in 293T cells (Fig. 6C), with the mass spectrum of HNRNPM and its fragments displayed in Fig. S4B. Endogenous Co-IP experiments in CRC cells further validated HNRNPM as an AURKB-binding partner (Fig. 6D). IF staining revealed the co-localization of AURKB and HNRNPM in CRC cells and tissues (Fig. 6E and Fig. S4C), and proximity ligation assays (PLA) further validated their physical interaction in clinical CRC specimens (Fig. 6F). Molecular docking simulations supported these findings, showing a strong binding energy of -22.8 kcal/mol between AURKB and HNRNPM (Fig. S4D), far exceeding the threshold of -5 kcal/mol for stable interactions.

To pinpoint the AURKB domain responsible for HNRNPM binding, full-length (FL) and truncated AURKB constructs (1-76aa, 1-251aa, 252-344aa) were co-expressed with HNRNPM. Co-IP assays revealed that only AURKB-FL and the 252-344aa truncation retained the ability to bind HNRNPM (Fig. 6G), indicating that HNRNPM selectively binds to the C-terminal domain (252-344aa) of AURKB. Notably, pharmacological inhibition of AURKB kinase activity with AZD2811 failed to disrupt its interaction with HNRNPM (Fig. S4E), which further supports a kinase-independent binding mechanism. Additionally, altering the expression of AURKB or HNRNPM left the expression of the other unaffected, suggesting that their interaction does not affect mutual protein stability or expression (Fig. S4F).

### AURKB inhibits HNRNPM-mediated PSAT1 mRNA degradation

**Fig. 7:**
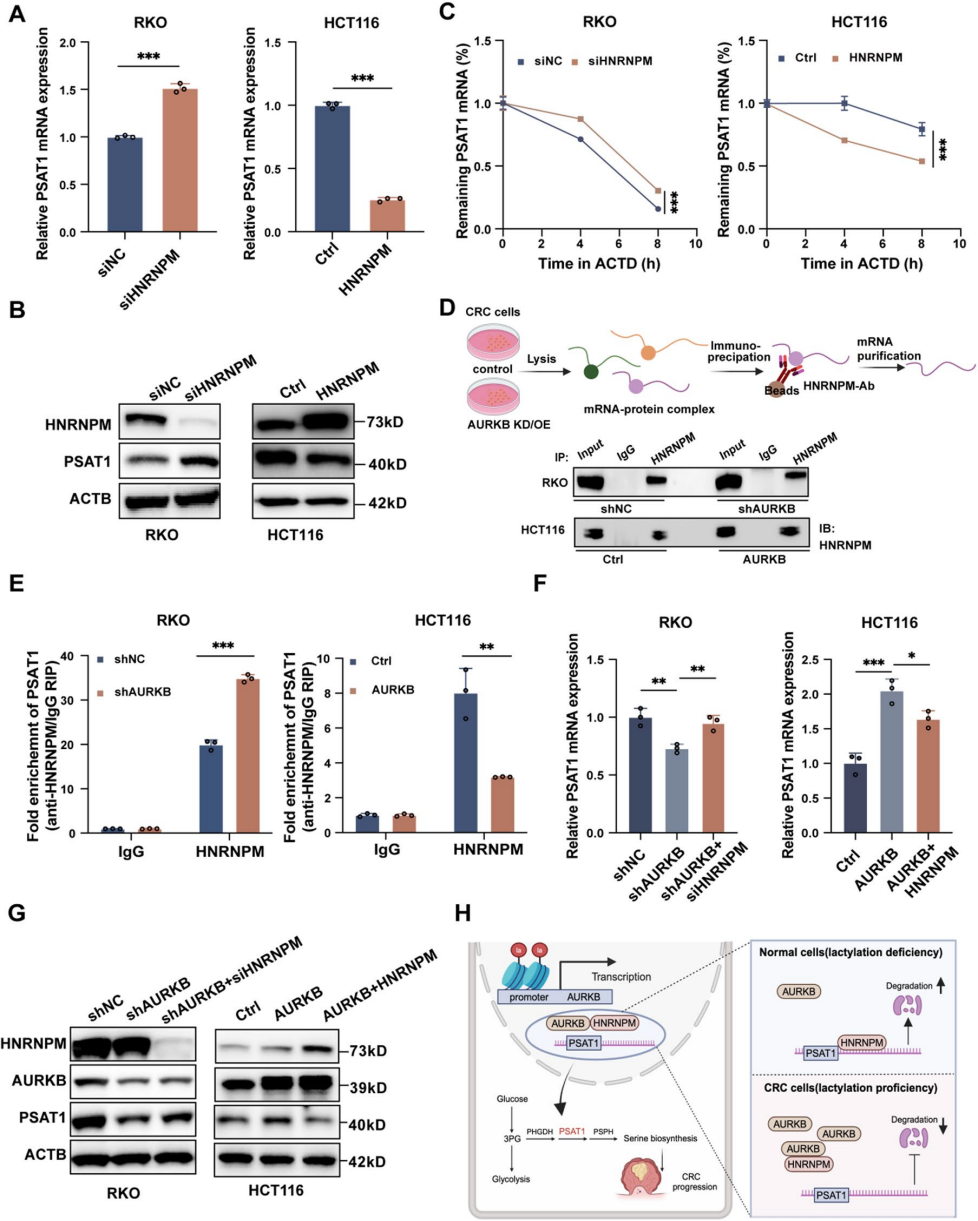
AURKB inhibits HNRNPM-mediated PSAT1 mRNA degradation. (**A-B**) PSAT1 mRNA (**A**) and protein (**B**) levels were measured in CRC cells following HNRNPM knockdown or overexpression, analyzed by RT-qPCR and western blot, respectively. (**C**) After transfection with siHNRNPM (#2) or an HNRNPM overexpression plasmid for 48 h, cells were treated with 5 µg/mL ACTD. Total RNA was extracted at 0 h, 4 h, and 8 h post-treatment, and PSAT1 mRNA levels were quantified by RT-qPCR. (**D**) RIP assay was performed to verify the direct interaction between HNRNPM and PSAT1 mRNA. Top: Schematic illustration of RIP procedure. Bottom: Representative western blot showing HNRNPM in the corresponding RIP elutes. (**E**) qRT-PCR analysis of RIP eluates to evaluate the enrichment of PSAT1 mRNA bound to HNRNPM following AURKB knockdown or overexpression. IgG served as a negative control. (**F-G**) The mRNA (**F**) and protein (**G**) levels of AURKB and PSAT1 in the indicated RKO and HCT116 cells. (**H**) Schematic summary of the proposed mechanism (created with BioRender). Data are presented as mean ± SD of three independent experiments in A, C, E and F. Statistical analysis was performed by two-tailed unpaired Student’s *t*-test in A, C and E, or by one-way ANOVA followed by Dunnett’s multiple comparisons test in F (*P < 0.05, **P < 0.01, and ***P < 0.001)

To determine whether AURKB regulates PSAT1 expression through its interaction with HNRNPM, we first interrogated the role of HNRNPM in regulating PSAT1 levels. As the results showed, both mRNA and protein expression of PSAT1 were markedly elevated following HNRNPM depletion and decreased by HNRNPM overexpression (Fig. 7A and B). In contrast, other HNRNPs showed no appreciable impact on PSAT1 expression (Fig. S5A and S5B). Consistent with the in vitro findings, xenograft tumors derived from HNRNPM-deficient cells also exhibited elevated PSAT1 levels, although tumor growth remained largely unaffected (Fig. S6A-D). HNRNPM is known to modulate alternative splicing [24–26] and mRNA degradation [27, 28], prompting us to investigate which mechanism underlies its effects on PSAT1 expression. PSAT1 encodes two isoforms (alpha and beta) [29], where the alpha isoform lacks exon 8, resulting in a 46-residue truncated protein (Fig. S7A). Isoform analysis displayed that the beta isoform predominates in CRC tissues (Fig. S7B). However, HNRNPM knockdown or overexpression did not alter the splicing pattern of PSAT1 (Fig. S7C and S7D), indicating that HNRNPM’s regulation of PSAT1 was not via HNRNPM-mediated alternative splicing. We then hypothesized that HNRNPM modulates PSAT1 mRNA stability. Actinomycin D chase assays demonstrated that HNRNPM depletion delayed PSAT1 mRNA degradation, whereas HNRNPM overexpression accelerated it (Fig. 7C). To further dissect the regulatory mechanism of HNRNPM, RIP followed by RT-qPCR analysis was performed. The results revealed a significant enrichment of PSAT1 mRNA precipitated by HNRNPM relative to IgG in CRC cells, with the binding efficiency appreciably increased by AURKB knockdown and decreased by AURKB overexpression (Fig. 7D and E). Furthermore, HNRNPM depletion partially rescued PSAT1 mRNA and protein suppression induced by AURKB knockdown. Conversely, overexpression of HNRNPM effectively mitigated the PSAT1 upregulation triggered by AURKB overexpression (Fig. 7F and G). Collectively, these findings suggest that AURKB inhibits the binding of HNRNPM to PSAT1 mRNA, thereby preventing its degradation and promoting PSAT1 mRNA and protein accumulation.

## Discussion

Although AURKB has long been recognized as a pan-cancer therapeutic target, the clinical efficacy of AURKB inhibitors has remained disappointing [13–17], raising concerns regarding non-classical, kinase-independent carcinogenic mechanisms. In the present study, we discovered a previously uncharacterized kinase-independent role of AURKB in regulating PSAT1 expression, which potentiated the oncogenic effects of AURKB in CRC. Mechanistically, we identified HNRNPM as a novel AURKB-binding partner that accelerates PSAT1 mRNA degradation; AURKB disrupts this interaction, thereby stabilizing PSAT1 transcripts and enhancing its expression (Fig. 7H).

Transcriptional upregulation of AURKB in malignancies has been conventionally attributed to oncogenic transcription factors such as c-Myc [10, 11], E2F1 [30], and Sp1 [31]. However, the epigenetic regulation of AURKB expression remains poorly understood. Histone lactylation, particularly H3K18la, has recently emerged as a key epigenetic modification linking lactate accumulation to gene activation [21, 32, 33]. In CRC, we found elevated levels of H3K18la and demonstrated its direct role in promoting AURKB transcription. Our findings replenish the understanding of how metabolic reprogramming rewires epigenetic landscapes to drive oncogene activation, underscoring the need for therapies that target both metabolic dysfunction and molecular effectors.

AURKB oncogenic activity has been attributed to its roles in cell cycle progression [6, 7, 34], apoptotic suppression [8, 9] and activation the PI3K/AKT signaling [35, 36]. RNA-seq and GSEA analysis revealed a link between AURKB and serine biosynthesis, aligning with prior transcriptomic data [11]. We identified PSAT1 as a pivotal downstream effector, corroborated by its known role in enhancing tumorigenic potential and resistance to oxaliplatin in CRC [37]. PSAT1 not only drives serine biosynthesis but also modulates key signaling pathways, including Hippo/YAP [38] and PI3K/AKT signaling cascade [39], highlighting its potential as a therapeutic target. Intriguingly, a recent study reported that PSAT1 depletion suppresses tumor growth only under exogenous serine deprivation [40], suggesting a compensatory role for nutrient uptake. Future research should explore whether dietary serine influences the therapeutic outcome of PSAT1-targeted strategies.

Notably, the suppressive effect of AURKB knockdown on PSAT1 expression was not recapitulated by treatment with AURKB inhibitors such as AZD2811, highlighting a kinase-independent regulatory pathway. This mirrors findings for AURKA, a close homolog of AURKB [41], which has been reported to facilitate tumor progression via modulation of alternative splicing [42]. Consistently, our proteomic and bioinformatics analysis revealed enrichment of spliceosome components and mRNA degradation pathways, raising the possibility that AURKB may interact with splicing factors to regulate the alternative splicing or mRNA stability of PSAT1.

HNRNPM was identified as a direct interacting partner of AURKB. As a highly conserved RNA-binding protein, HNRNPM plays a pivotal role in post-transcriptional regulation. Specifically, it modulates CD44 alternative splicing, favoring the conversion from variant isoforms (CD44v) to the standard isoform (CD44s), which drives epithelial-mesenchymal transition (EMT) and enhances invasion and metastasis in triple-negative breast cancer [24, 43, 44]. Several studies have highlighted that HNRNPM, in coordination with non-coding RNAs, regulates the stability of target mRNAs, thereby activating downstream oncogenic signaling [27, 28]. However, the role of HNRNPM in tumor progression appears to be cancer-type specific and highly context-dependent. For instance, loss of HNRNPM has been shown to induce splicing perturbations and contribute to lung cancer pathogenesis [26], whereas in CRC, its function is largely determined by the SUMOylation status of lysine 17 (K17), with the SUMOylated form impeding cancer cell glycolysis and tumorigenesis [45]. In our study, knockdown of HNRNPM in HCT116 xenograft models led to increased PSAT1 expression, yet had no observable impact on tumor growth. This finding suggests that the pleiotropic and context-specific roles of HNRNPM may involve additional mechanisms beyond PSAT1 regulation, necessitating further exploration. Subsequent mechanistic analyses demonstrated that HNRNPM directly promotes the degradation of PSAT1 mRNA, independent of alternative splicing regulation. Notably, the kinase-independent interaction between AURKB and HNRNPM does not alter the expression level of HNRNPM but compromises its binding affinity to PSAT1 mRNA, thereby reducing mRNA turnover and promoting PSAT1 protein accumulation.

Although kinase inhibitors targeting AURKB have yielded modest results clinically, they may yet hold promise in combinatorial approaches with chemotherapy, radiotherapy, or targeted agents [8, 46]. Moreover, board-spectrum inhibitors such as chiauranib, which concurrently target AURKB, CSF1R, and VEGFR, offer a strategy to overcome the limitations of AURKB-selective inhibitors [47, 48]. Our study demonstrates that AURKB facilitates CRC progression through kinase-independent regulation of PSAT1. This provides novel perspectives for AURKB-targeted therapy, suggesting that, beyond enzymatic inhibition, strategies such as protein degraders (e.g., PROTACs) or pharmacological inhibition of the AURKB/PSAT1 axis should also be explored.

## Conclusion

In summary, our study reveals that AURKB is markedly upregulated in CRC and correlates with disease progression and poor prognosis. H3K18la epigenetically activates AURKB transcription, and elevated AURKB subsequently disrupts HNRNPM-mediated PSAT1 mRNA degradation, stabilizing PSAT1 expression and promoting tumor growth. These findings redefine AURKB’s oncogenic role beyond its kinase activity and provide novel mechanistic insight into CRC progression, with broad implications for therapeutic development.

## Supplementary Information

Below is the link to the electronic supplementary material.

Supplementary Material 1

Supplementary Material 2

Supplementary Material 3

## Data Availability

The RNA sequencing data have been deposited in the GEO database under accession number GSE292281.

## Abbreviations

CRC: Colorectal cancer
TCGA: The Cancer Genome Atlas
AURKB: Aurora kinase B
GEO: Gene Expression Omnibus
FDR: False Discovery Rate
GSEA: Gene Set Enrichment Analysis
ChIP: Chromatin immunoprecipitation
HNRNPM: Heterogeneous nuclear ribonucleoprotein M
PHGDH: Phosphoglycerate dehydrogenase
PSAT1: Phosphoserine aminotransferase 1
PSPH: Phosphoserine phosphatase
RIP: RNA immunoprecipitation
H3K18la: Histone H3 lysine 18 lactylation
ACTB: β-actin
qPCR: Quantitative PCR
SD: Standard deviation
OS: Overall survival
PFS: Progression free survival
PLA: Proximity ligation assay
