# Supplementary material for: Histone lactylation-boosted AURKB facilitates colorectal cancer progression by inhibiting HNRNPM-mediated PSAT1 mRNA degradation
Source: J Exp Clin Cancer Res. 2025 Aug 11;44:233. doi: 10.1186/s13046-025-03498-1 (PMC12337393; doi:10.1186/s13046-025-03498-1)
Supplement: Supplementary file 1 — Supplementary Material 1 [file 13046_2025_3498_MOESM1_ESM.pdf]

上海交通大学医学院附属第九人民医院动物伦理委员会伦理审查批准函

Ethical Review Approval of the Laboratory Animal Ethics Committee in Ninth People's Hospital Affiliated to Shanghai Jiao Tong University School of Medicine

批件号 Approval No.：SH9H-2023-A775-1

<table>
<tr><td rowspan="4">课题信息
Protocol Information</td><td>项目课题名称
Project Title</td><td colspan="4">AURKB对结直肠癌的影响及机制研究</td></tr>
<tr><td>项目来源及编号
Sponsor and Grant No.</td><td colspan="2">☐863 项目 ☐973 项目 ☐国家专项
■国自然 ☐上海市项目 ☐非国家专项
☐其他：</td><td>课题负责人
Principle Investigator</td><td>孟祥军</td></tr>
<tr><td>研究单位/科室
Department</td><td>上海交通大学医学院附属第九人民医院/消化内科</td><td colspan="2">拟动物实验起止时间
Period of Animal Experiment</td><td>2023 年 01 月 01 日
2025 年 01 月 01 日</td></tr>
<tr><td>动物品种/品系
Breed/Strain</td><td>BALB/c 裸鼠</td><td colspan="2">动物数量/只
Quantity</td><td>100</td></tr>
<tr><td rowspan="3">审查信息
Review Information</td><td>审查日期
Review Date</td><td>2023 年 06 月 20 日</td><td colspan="2" rowspan="2">审查方式
Method of Review</td><td rowspan="2">☐会议审查
Review meeting
■线上审查
Online review</td></tr>
<tr><td>审查委员
Reviewer</td><td>许锋</td></tr>
<tr><td>审查结果
Results</td><td colspan="4">1. 经本机构伦理委员会审查，审查意见和建议：
Review comments and recommendations by the Ethics Committee:
■同意 Agree
☐修改后同意 Agree after modify
☐修改后重审 Re-review after modify
☐不同意 Disagree
☐终止或暂停已批准的实验 Terminate or suspend approved experiments
2. 机构伦理委员会对该研究实施过程的定期跟踪审查：☐是 Yes ■否 No
A periodic follow-up review of the research implementation process by the Ethics Committee
☐3 个月 per 3 months ☐6 个月 per 6 months ☐12 个月 per 12 months
3. 自批准之日起，该批件有效期为 3 年。
The approval is valid for three years from the date of approval.</td></tr>
</table>

声明 Statement	本机构伦理委员会按照国家《实验动物福利伦理审查指南》组成和工作，其审查和工作过程不受机构伦理委员会以外任何组织及个人的影响。<br>The Ethics Committee is formed and operated in accordance with Chinese National Standard *Laboratory animal—Guideline for ethical review of animal welfare*. The review and working process of members shall not be influenced by any organization or individual other than the institutional Ethics Committee.<br>本项目动物实验方案经实验动物伦理委员会审核，符合国家实验动物福利伦理的相关规定。<br>The animal use protocol has been reviewed and approved by the Laboratory Animal Ethics Committee and is in accordance with the relevant provisions of the National Experimental Animal Welfare Ethics.

主任委员签名 Signature of Chairman：

伦理委员会（盖章） Seal of Ethics Committee

日期 Date 2023 年 06 月 21 日

地址：上海市制造局路 639 号 邮编：200011 电话：021-23271699*5576
Address: No.639 Zhizaoju Road, Shanghai, P.R China Postcode: 200011 Tel: +86-021-23271699*5576
