# Supplementary material for: Histone lactylation-boosted AURKB facilitates colorectal cancer progression by inhibiting HNRNPM-mediated PSAT1 mRNA degradation
Source: J Exp Clin Cancer Res. 2025 Aug 11;44:233. doi: 10.1186/s13046-025-03498-1 (PMC12337393; doi:10.1186/s13046-025-03498-1)
Supplement: Supplementary file 2 — Supplementary Material 2 [file 13046_2025_3498_MOESM2_ESM.docx]

**Table S1 The detailed information of clinical samples used in this study.**

| **Case** | **Location** | **Gender** | **Age** | **Grade** | **Pathology** |
| --- | --- | --- | --- | --- | --- |
| CRC1 | proximal | Male | 80 | Moderately differentiated | Adenocarcinoma |
| CRC2 | Rectum | Female | 64 | Moderately differentiated | Adenocarcinoma |
| CRC3 | distal | Male | 60 | Moderately differentiated | Adenocarcinoma |
| CRC4 | distal | Male | 80 | Moderately differentiated | Adenocarcinoma |
| CRC5 | distal | Male | 71 | Moderately differentiated | Adenocarcinoma |
| CRC6 | proximal | Male | 73 | Moderately differentiated | Adenocarcinoma |
| CRC7 | distal | Male | 84 | Well differentiated | Adenocarcinoma |
| CRC8 | Rectum | Female | 69 | Moderately differentiated | Adenocarcinoma |
| CRC9 | Rectum | Male | 64 | Moderately differentiated | Adenocarcinoma |
| CRC10 | Rectum | Female | 52 | Moderately differentiated | Adenocarcinoma |
| CRC11 | Rectum | Male | 71 | Moderately differentiated | Adenocarcinoma |
| CRC12 | proximal | Female | 52 | Moderately differentiated | Adenocarcinoma |
| CRC13 | Rectum | Female | 87 | Moderately differentiated | Adenocarcinoma |
| CRC14 | Rectum | Female | 63 | Moderately differentiated | Adenocarcinoma |
| CRC15 | Rectum | Male | 73 | Moderately differentiated | Adenocarcinoma |
| CRC16 | Rectum | Male | 68 | Moderately differentiated | Adenocarcinoma |
| CRC17 | Rectum | Male | 71 | Moderately differentiated | Adenocarcinoma |
| CRC18 | Rectum | Male | 74 | Moderately differentiated | Adenocarcinoma |
| CRC19 | distal | Female | 52 | Moderately differentiated | Adenocarcinoma |
| CRC20 | proximal | Female | 80 | Moderately differentiated | Adenocarcinoma |
| CRC21 | distal | Male | 80 | Moderately differentiated | Adenocarcinoma |
| CRC22 | proximal | Male | 74 | Moderately differentiated | Adenocarcinoma |
| CRC23 | proximal | Female | 69 | Moderately differentiated | Adenocarcinoma |
| CRC24 | distal | Male | 71 | Moderately differentiated | Adenocarcinoma |
| CRC25 | proximal | Male | 51 | Moderately differentiated | Adenocarcinoma |

WD: well-differentiated; MD: moderate-differentiated.

**Table S2 Antibodies used in this study.**

| Antibody | Source | Application | Catalog No. |
| --- | --- | --- | --- |
| AURKB (Mouse) | Santa Cruz Biotechnology | WB | #sc393357 |
| AURKB (Rabbit) | Abclonal | PLA, IF, IP, IHC | #A19539 |
| Caspase 3 | Cell Signaling Technology | WB | #9662 |
| Cleaved Caspase 3 | Cell Signaling Technology | WB, IHC | #9661 |
| Flag (Mouse) | Abmart | WB | #M20008 |
| Flag (Rabbit) | Abmart | IP | #T20008 |
| H3 | Abmart | WB | #TD6932 |
| H3K18la | PTM BIO | ChIP, WB, IHC | #PTM-1427RM |
| IgG | Cell Signaling Technology | IP | #2729 |
| HA | Santa Cruz Biotechnology | WB | #sc-57592 |
| HNRNPM (Mouse) | Santa Cruz Biotechnology | WB, PLA, IF | #sc-20001 |
| HNRNPM (Rabbit) | Abclonal | IP | #T57256 |
| Ki67 | Abmart | IHC | #A20018 |
| LDHA | Santa Cruz Biotechnology | WB | #sc133123 |
| LDHB | Proteintech | WB | #14824-1-AP |
| p53 | Abmart | WB | #TA0879 |
| PanKla | PTM BIO | WB, IHC | #PTM-1401RM |
| PHGDH | Abclonal | WB | #A10461 |
| PSAT1 | Proteintech | WB, IHC | #10501-1-AP |
| PSPH | Abclonal | WB | #A22763 |
| pH3Ser10 | STARTER | WB | #S0B1410 |

**Table S3 Primers, siRNA and shRNA sequences in this study.**

| **Purpose** | **Names** | **Sequence (5′-3′)** |
| --- | --- | --- |
| qPCR | HNRNPM-F | CTCTTAATGGACGCTGAAGGAAA |
|  | HNRNPM-R | CGCTCAGACTATGCTTGTTTAGG |
|  | PHGDH-F | GAATGATCATGTGCCTGGC |
|  | PHGDH-R | GTTCCCATGAACTTCTTCCG |
|  | PSAT1-F | TGCCGCACTCAGTGTTGTTAG |
|  | PSAT1-R | GCAATTCCCGCACAAGATTCT |
|  | PSPH-F | GAGGACGCGGTGTCAGAAAT |
|  | PSPH-R | GGTTGCTCTGCTATGAGTCTCT |
|  | AURKB-F | CAGTGGGACACCCGACATC |
|  | AURKB-R | GTACACGTTTCCAAACTTGCC |
|  | GAPDH-F | ACAACTTTGGTATCGTGGAAGG |
|  | GAPDH-R | GCCATCACGCCACAGTTTC |
| semi-quantitative PCR | PSAT1-AS-F | CAGCATCTACGTCATGGGCT |
|  | PSAT1-AS-R | TTTTTCATGAAGGCGGCCAG |
| ChIP-qPCR | AURKB-F | CACTGGGGGAATTTGGGGAA |
|  | AURKB-R | TTGGCTCGATGTCCTGTGAC |
| siRNA assay | siNC | UUCUCCGAACGUGUCACGU |
|  | siAURKB#1 | CCUGCGUCUCUACAACUAU |
|  | siAURKB#2 | UCGUCAAGGUGGACCUAAA |
|  | siLDHA | CCAGUUUCCACCAUGAUUA |
|  | siLDHB | GGAUAUACCAACUGGGCUA |
|  | siHNRNPM#1 | GCAUCGGAAUGGGAAACAU |
|  | siHNRNPM#2 | CCAUUUGACUGUUUGCAUU |
| shRNA assay | shNC | CCUAAGGUUAAGUCGCCCTCG |
|  | shAURKB | CCUGCGUCUCUACAACUAU |
|  | shHNRNPM | GGCAUAGGAUUUGGAAUAA |

**Table S4 Correlation of AURKB expression and clinicopathologic/molecular characteristics in CRC patients.**

|  |  | **AURKB level** | |  |
| --- | --- | --- | --- | --- |
| **Characteristics** | **N** | **Low** | **High** | ***P*** |
| **Total cases** | 585 | 468 | 117 |  |
| **Gender** |  |  |  | 0.934 |
| Male | 322 | 258 | 64 |  |
| Female | 263 | 210 | 53 |  |
| **Age** |  |  |  | 0.390 |
| ＜60 | 153 | 126 | 27 |  |
| >=60 | 431 | 341 | 90 |  |
| **Location** |  |  |  | 0.165 |
| Proximal | 232 | 192 | 40 |  |
| Distal | 351 | 274 | 77 |  |
| **TNM stage** |  |  |  | **0.017*** |
| I/II | 309 | 258 | 51 |  |
| III/IV | 270 | 204 | 66 |  |
| **T stage** |  |  |  | 0.381 |
| Tis/T1/T2 | 64 | 54 | 10 |  |
| T3/T4 | 498 | 398 | 100 |  |
| **N stage** |  |  |  | **0.014*** |
| N0 | 314 | 264 | 50 |  |
| N1/N2/N3 | 243 | 184 | 59 |  |
| **M stage** |  |  |  | 0.620 |
| M0 | 499 | 403 | 96 |  |
| M1 | 61 | 48 | 13 |  |
| **CIN** |  |  |  | 0.087 |
| - | 112 | 96 | 16 |  |
| + | 369 | 289 | 80 |  |
| **CIMP** |  |  |  | 0.630 |
| - | 420 | 334 | 86 |  |
| + | 93 | 76 | 17 |  |
| **MMR** |  |  |  | 0.436 |
| pMMR | 459 | 364 | 95 |  |
| dMMR | 77 | 64 | 13 |  |
| **TP53** |  |  |  | 0.419 |
| WT | 161 | 127 | 34 |  |
| M | 190 | 148 | 42 |  |
| **KRAS** |  |  |  | 0.123 |
| WT | 328 | 266 | 62 |  |
| M | 217 | 165 | 52 |  |
| **BRAF** |  |  |  | 0.170 |
| WT | 461 | 362 | 99 |  |
| M | 51 | 44 | 7 |  |

WT: wild-type; M: mutant; CIN: chromosomal instability; CIMP: CpG island methylator phenotype; MMR: mismatch repair.

**Table S5 The fold changes of genes involved in serine and glycine metabolism.**

|  | **shAURKB vs shNC**  **（RKO cells）** | |  | **AURKB_high vs AURKB_low （TCGA-COAD）** | |
| --- | --- | --- | --- | --- | --- |
| **Gene** | **log2FC** | **FDR** |  | **log2FC** | **FDR** |
| SARDH | -2.335348525 | 0.005846183 |  | -0.275990287 | 0.044145335 |
| PIPOX | -1.689984317 | 0.291348466 |  | -0.08095133 | 0.755684638 |
| CTH | -1.359320488 | 2.37768E-83 |  | 0.03110717 | 0.815054114 |
| **PSAT1** | -1.149287603 | 2.19059E-21 |  | 0.786075313 | 2.66855E-08 |
| GLDC | -1.0896695 | 0.435676579 |  | 0.478733725 | 0.065893292 |
| GATM | -1.066582022 | 5.21939E-05 |  | -0.688277544 | 0.000120154 |
| **PSPH** | -0.886684152 | 4.7381E-29 |  | 0.407170968 | 0.000115523 |
| MAOA | -0.594201883 | 0.716363644 |  | -0.385984135 | 0.017107951 |
| **PHGDH** | -0.455563216 | 1.59418E-20 |  | 0.638193814 | 0.00103505 |
| SRR | -0.444980618 | 0.000529205 |  | 0.082048126 | 0.344724146 |
| GCAT | -0.388806281 | 9.82204E-06 |  | 0.429839131 | 0.001443137 |
| GLYCTK | -0.155905911 | 0.227923638 |  | -0.255209557 | 0.04730085 |
| AMT | -0.09941786 | 0.682673792 |  | -0.97016443 | 1.93886E-09 |
| CHDH | -0.00293628 | 0.983391772 |  | 0.002636885 | 0.982122009 |
| DMGDH | 0.005939008 | 0.995518706 |  | -0.632847249 | 0.000252874 |
| ALAS1 | 0.085973563 | 0.189931553 |  | 0.057036361 | 0.50602907 |
| SHMT2 | 0.13658777 | 0.000575575 |  | 0.526295335 | 1.49944E-06 |
| DLD | 0.260780078 | 2.46504E-12 |  | 0.048233946 | 0.477116989 |
| SHMT1 | 0.305857599 | 3.07856E-05 |  | 0.365499444 | 5.43672E-05 |
| AOC2 | 0.319071688 | 0.097766092 |  | -0.123843649 | 0.473086068 |
| AOC3 | 0.98733592 | 7.9237E-05 |  | -1.192157679 | 6.53371E-09 |
