# Supplementary material for: Histone lactylation-boosted AURKB facilitates colorectal cancer progression by inhibiting HNRNPM-mediated PSAT1 mRNA degradation
Source: J Exp Clin Cancer Res. 2025 Aug 11;44:233. doi: 10.1186/s13046-025-03498-1 (PMC12337393; doi:10.1186/s13046-025-03498-1)
Supplement: Supplementary file 3 — Supplementary Material 3 [file 13046_2025_3498_MOESM3_ESM.docx]

**Supplementary figure legend**

**
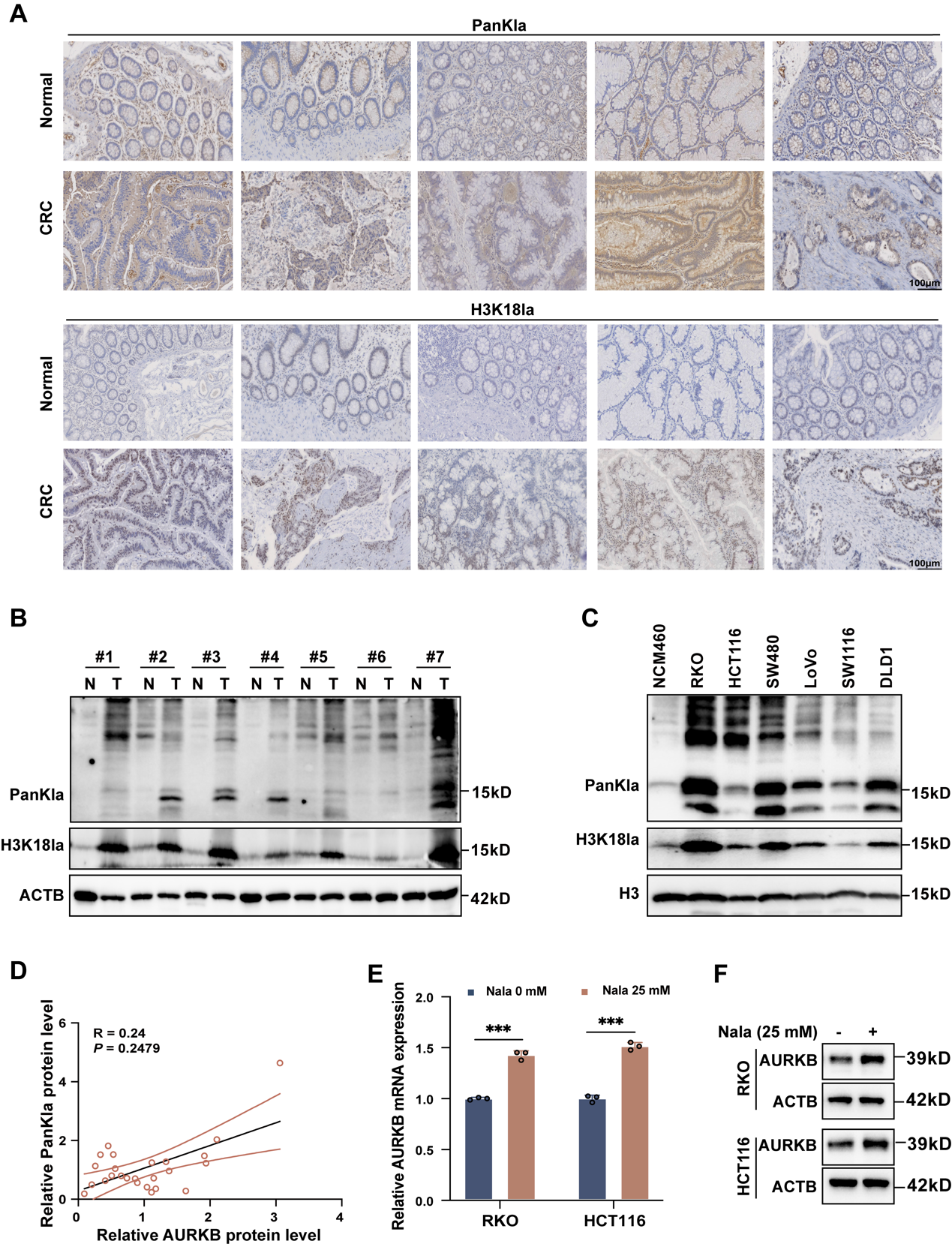
**

**Fig. S1 H3K18la promotes AURKB transcription in CRC.** (A-B) The expression of PanKla and H3K18la in CRC and paired normal tissues were examined by IHC (n = 5 pairs, scale bar = 100μm) and western blot assay (n = 7 pairs; N: normal, T: CRC). (C) Western blot analysis of PanKla and H3K18la levels in normal colonic epithelial cells and CRC cell lines. (D) Correlation analysis between the relative expression levels of PanKla and AURKB (n = 25). (E-F) AURKB mRNA (E) and protein (F) levels were measured in RKO and HCT116 cells after 48 h treatment with 25 mM Nala. Data are shown as mean ± SD of three independent experiments in E. Statistical analysis was performed by two-sided Spearman’s correlation test in D, or by two-tailed unpaired Student's *t*-test in E (**P* < 0.05, ***P* < 0.01, and ****P* < 0.001).


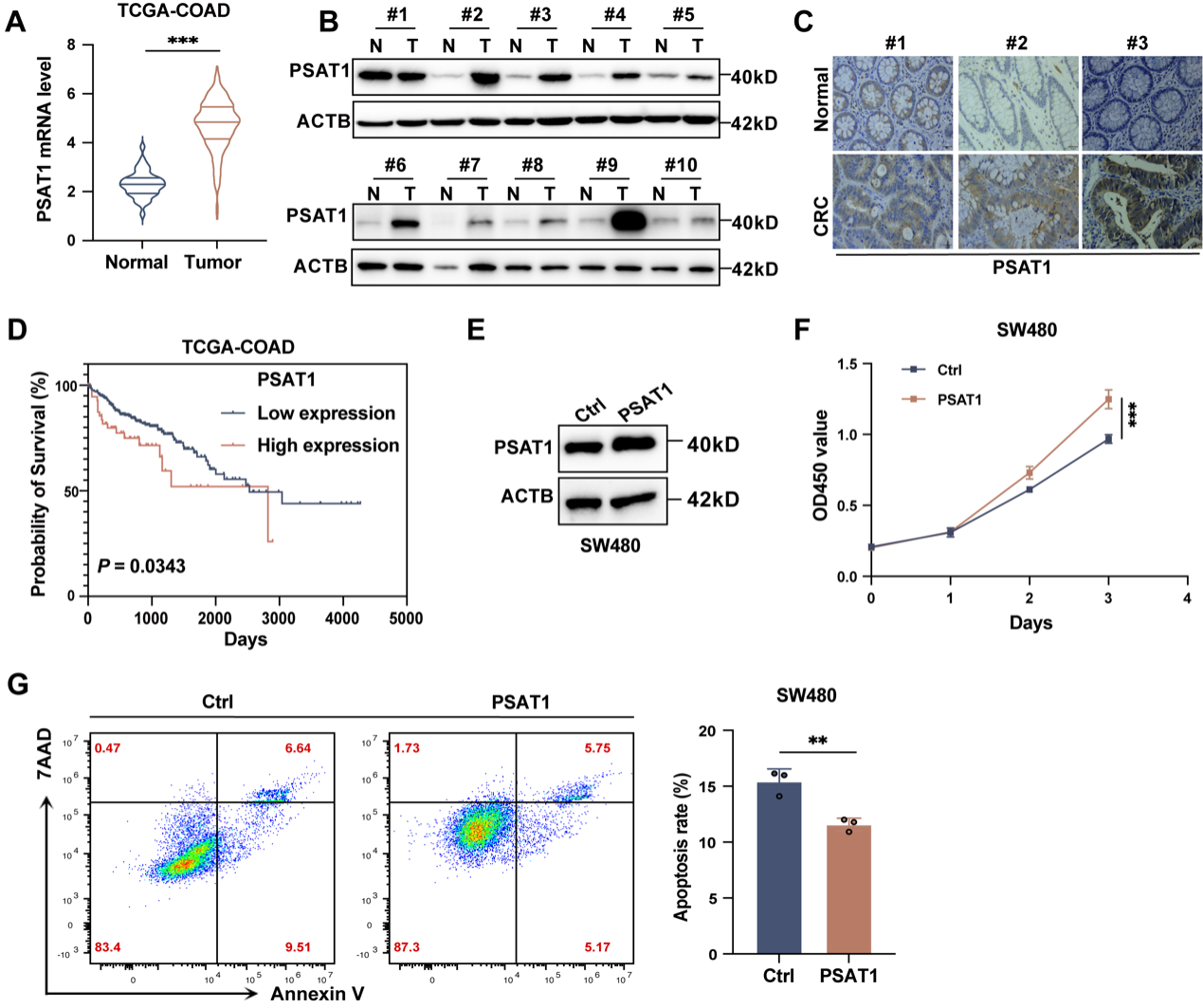


**Fig. S2 Functional role of PSAT1 in CRC.** (A) PSAT1 mRNA levels in CRC (n = 453) and normal tissues (n = 51) from the TCGA-COAD cohort. (B-C) PSAT1 protein expression in CRC and paired normal tissues (n = 10 pairs; N: normal, T: CRC) and IHC assay (n = 3 pairs, scale bar = 10μm). (D) Kaplan-Meier analysis showing the correlation between PSAT1 expression and overall survival of patients from the TCGA-COAD cohort (n = 453). (E) PSAT1 overexpression efficiency was validated by western blot. (F-G) CCK-8 and flow cytometry assays to evaluate cell proliferation (F) and apoptosis (G) in SW480 cells following PSAT1 overexpression. Data are presented as mean ± SD of three independent experiments in F and G. Statistical significance was determined by two-tailed unpaired Student's *t*-test in A, F, and G, or by the log-rank test in D (**P* < 0.05, ***P* < 0.01, and ****P* < 0.001).


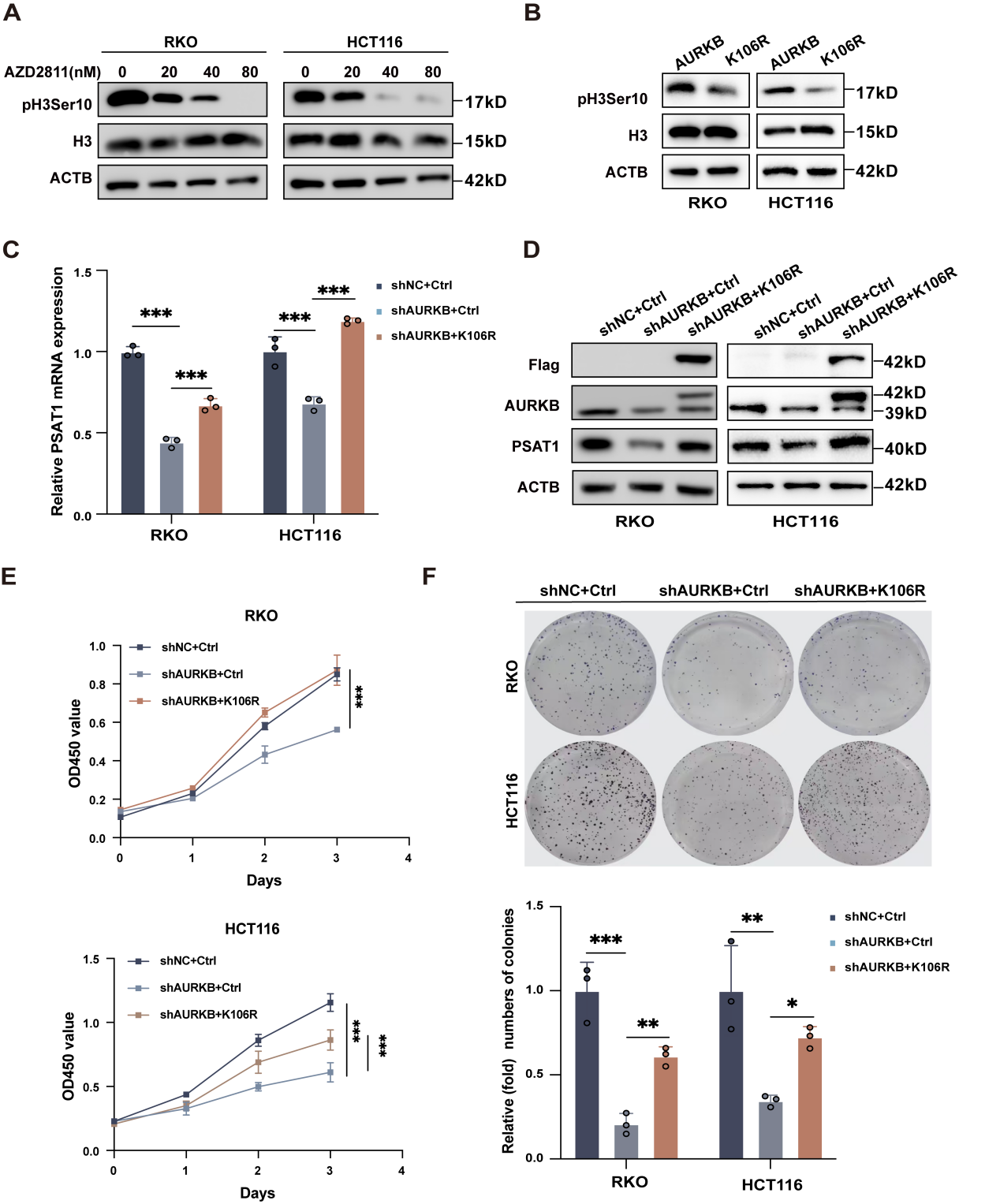


**Fig. S3 AURKB regulates PSAT1 expression in a kinase activity-independent manner.** (A) Western blot analysis showing the levels of histone H3 phosphorylation at serine 10 (pH3Ser10) in RKO and HCT116 cells following treatment with increasing concentrations of AZD2811. (B) Validation of pH3Ser10 levels in HCT116 and RKO cells transfected with either wild-type AURKB or the kinase-dead mutant K106R, as assessed by western blot. (C-D) mRNA (C) and protein (D) expression levels of PSAT1 in AURKB-knockdown CRC cells with or without ectopic expression of the kinase-dead AURKB mutant (K106R). (E-F) Cell proliferation capacity evaluated by CCK-8 (E) and colony formation assay (F) in AURKB-depleted CRC cells transfected with or without K106R. Data are presented as mean ± SD of three independent experiments in C, E, and F. Statistical significance was determined by one-way ANOVA followed by Dunnett’s multiple comparisons test (**P* < 0.05, ***P* < 0.01, and ****P* < 0.001).


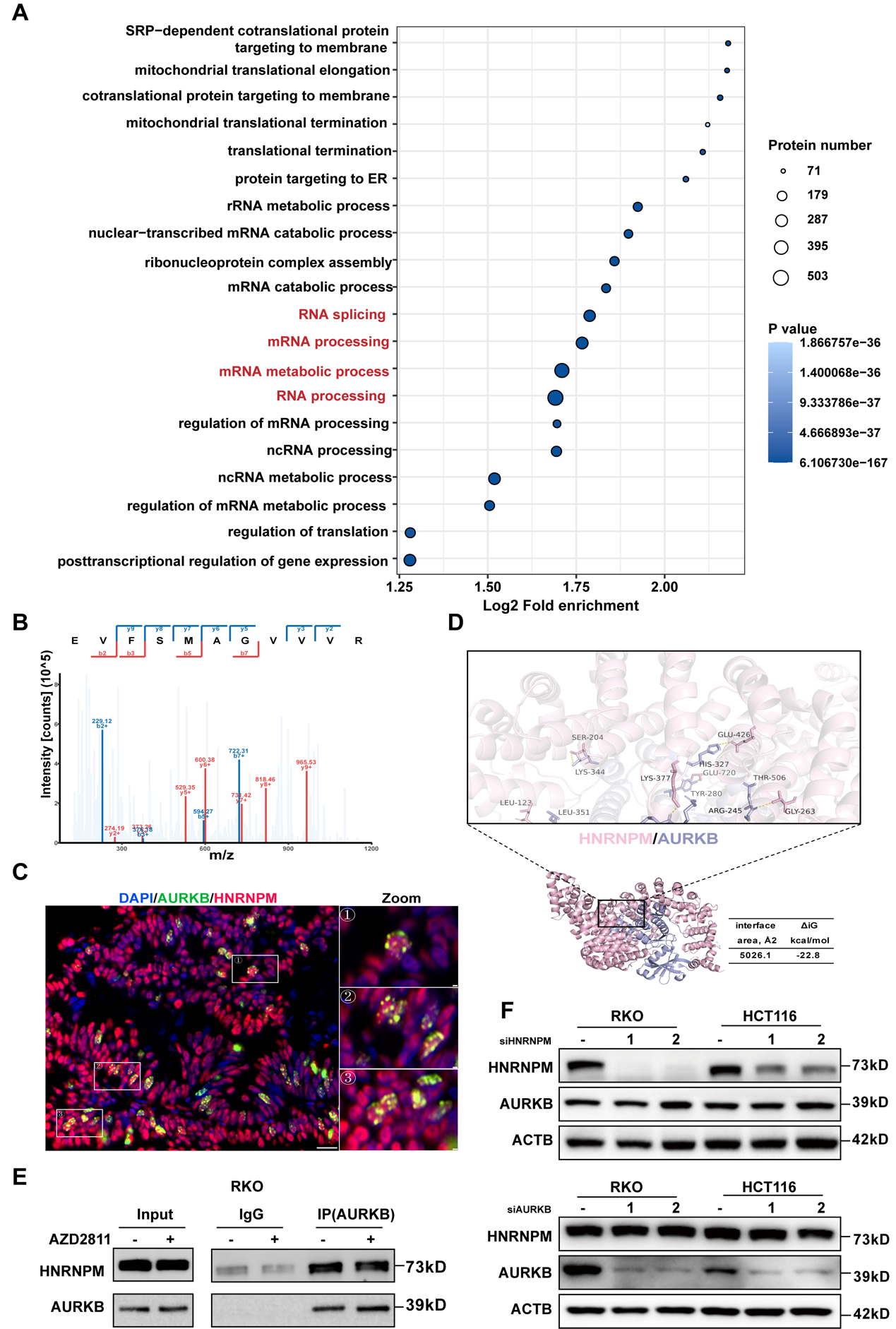


**Fig. S4 AURKB interacts with HNRNPM.** (A) GO enrichment analysis of AURKB-interacting proteins identified by IP-MS in 293T cells. (B) Immunoprecipitation of exogenous AURKB in 293T cells using an anti-Flag antibody, followed by MS analysis, confirmed the presence of peptide fragments of HNRNPM. (C) Representative fluorescence images showing the co-localization of AURKB (green) and HNRNPM (red) in CRC tissues (scale bar = 20μm; zoomed inset = 2μm), with nuclei counterstained with DAPI (blue). (D) Structural analysis of the protein-protein interaction interface between AURKB (purple ribbon structure) and HNRNPM (red ribbon structure). (E) Co-IP assay validating the interaction between AURKB and HNRNPM in RKO cells treated with or without 80 nM AZD2811. (F) Western blot analysis of AURKB and HNRNPM expression levels following AURKB knockdown or HNRNPM knockdown.


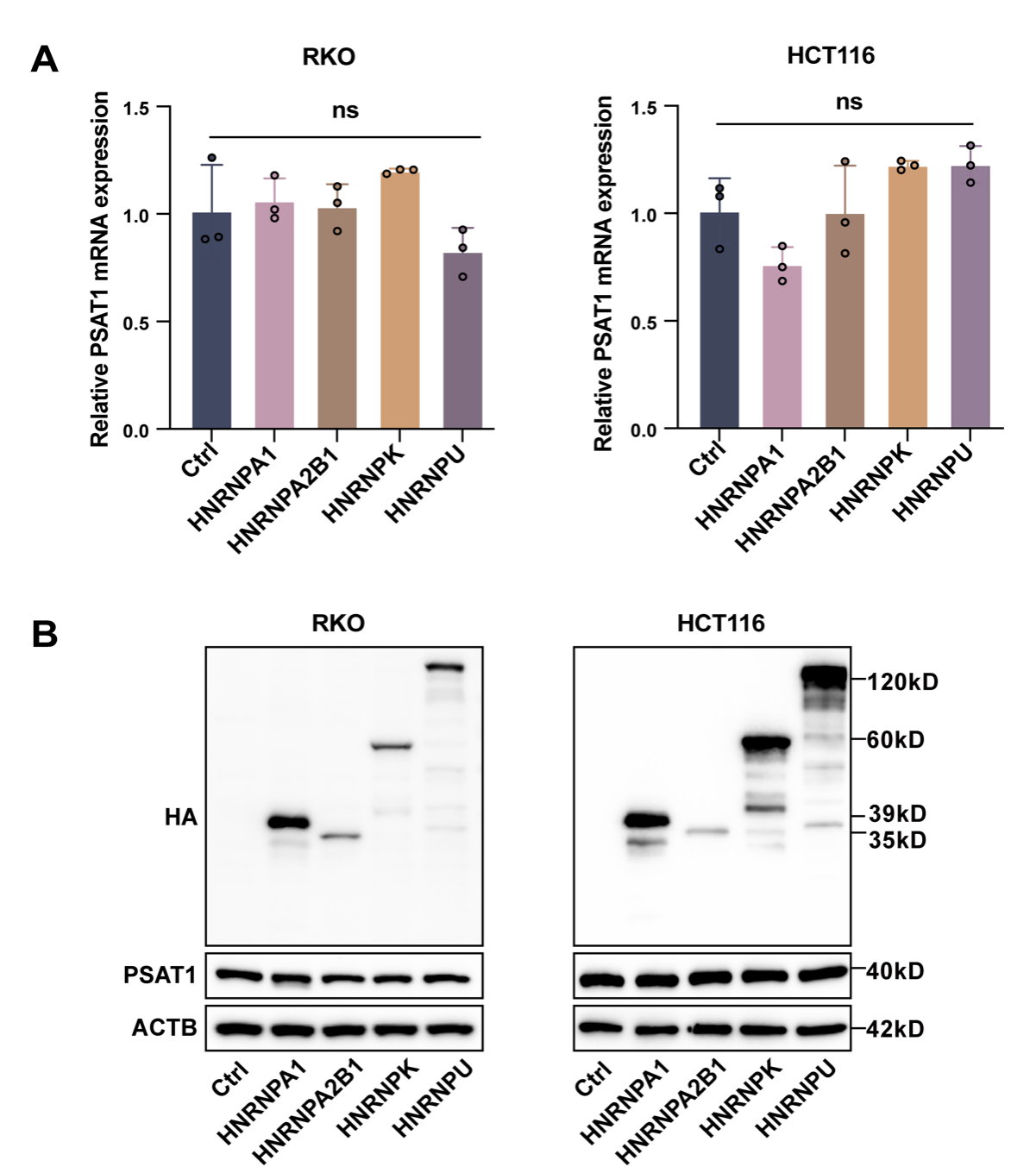


**Fig. S5 The effects of HNRNPs on PSAT1 expression.** (A-B) PSAT1 mRNA (A) and protein (B) levels in HCT116 and RKO cells after transfection with HA-tagged HNRNPs plasmids for 48 h. Data are presented as mean ± SD of three independent experiments in A. Statistical significance was determined by one-way ANOVA followed by Dunnett’s multiple comparisons test (**P* < 0.05, ***P* < 0.01, and ****P* < 0.001).


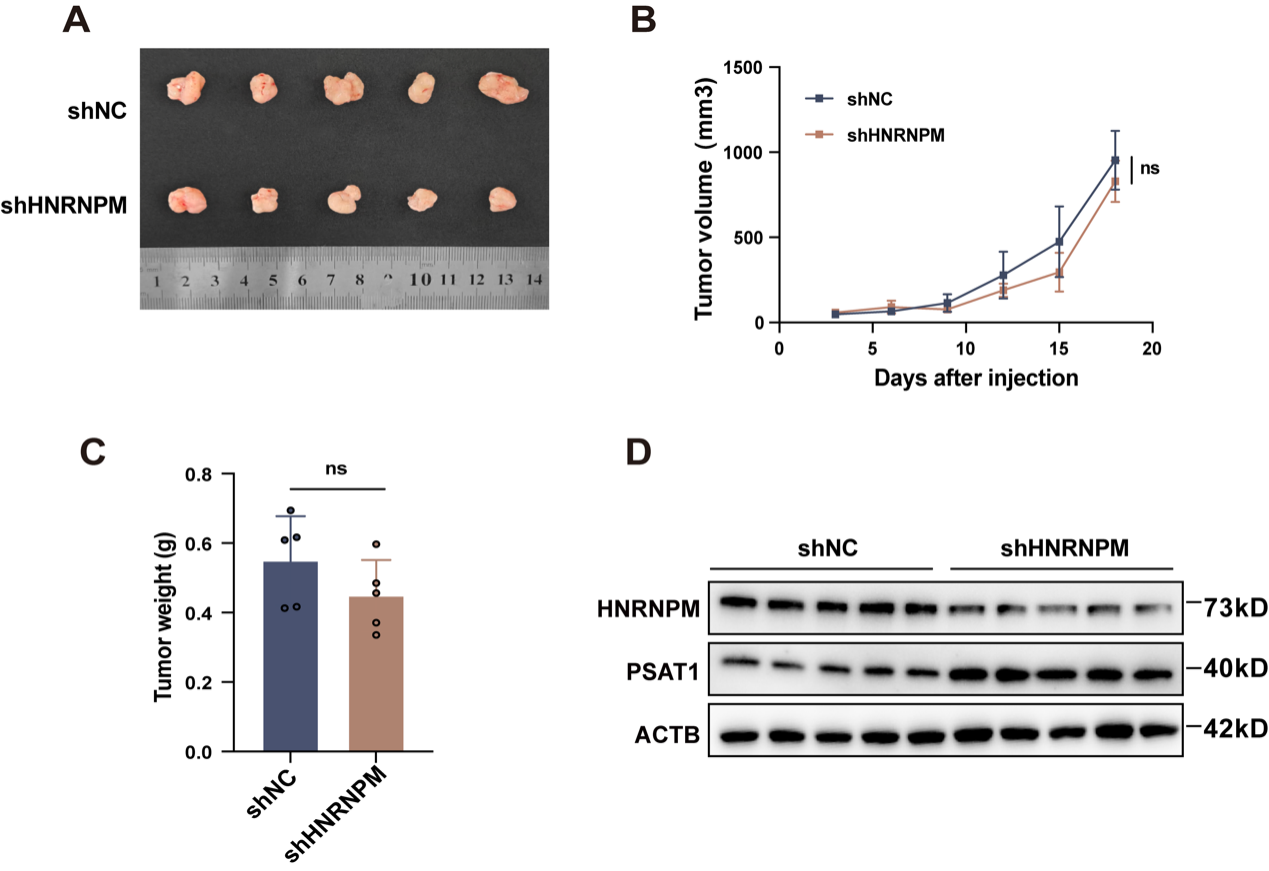


**Fig. S6 *In vivo* validation of the HNRNPM/PSAT1 regulatory axis.** (A-C) HCT116 cells stably expressing shNC or shHNRNPM were subcutaneously implanted into nude mice (n = 5 per group). Representative tumor images (A), tumor growth curves (B), and tumor weights (C) were recorded and analyzed. Data are shown as mean ± SD and analyzed by two-tailed unpaired Student's *t*-test (**P* < 0.05, ***P* < 0.01, and ****P* < 0.001; ns, *P* > 0.05). (D) Western blot analysis of HNRNPM and PSAT1 expression in excised xenograft tumor tissues.


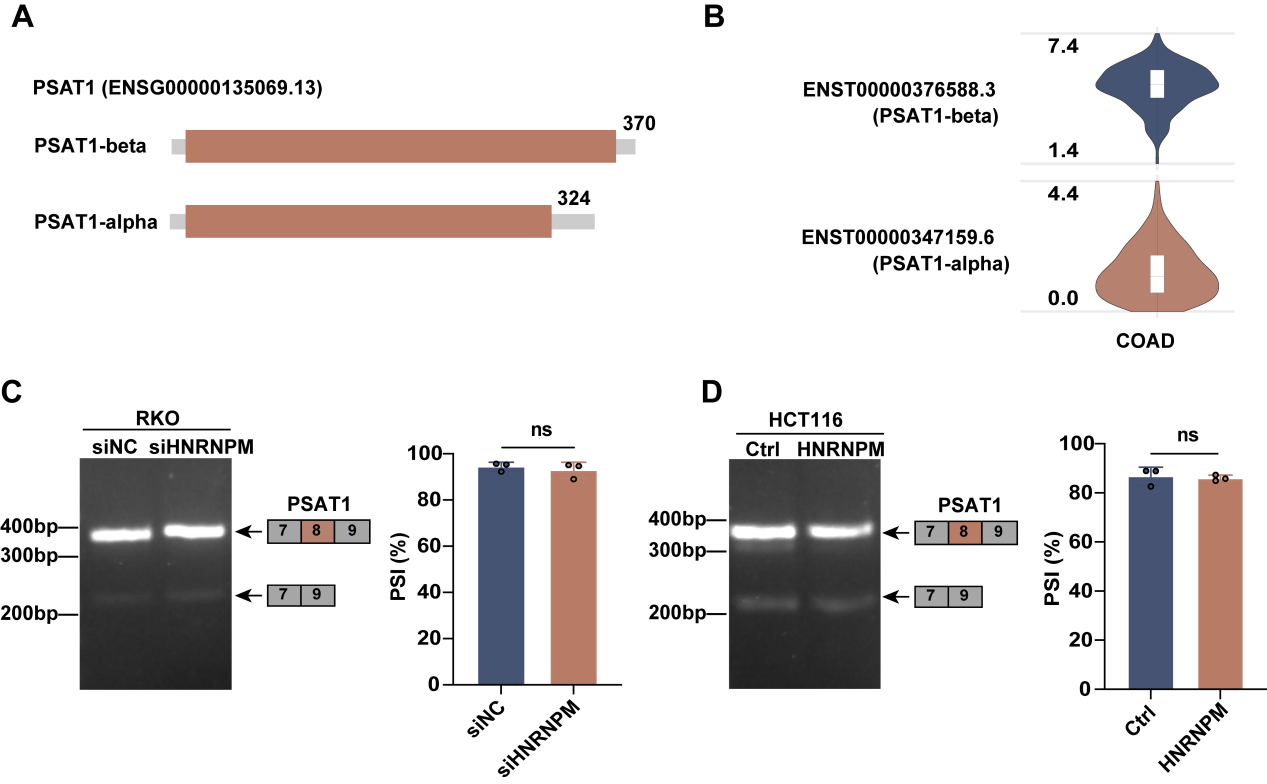


**Fig. S7 Regulation of PSAT1 by HNRNPM is independent of alternative splicing.**

(A) Schematic representation of PSAT1 isoform structures. (B) Expression profiles of PSAT1 isoforms in CRC tissues from the TCGA-COAD dataset were analyzed using the GEPIA2 online platform (<http://gepia2.cancer-pku.cn>). (C-D) Representative gel images of semi-quantitative RT-PCR products and the corresponding percent spliced-in (PSI) values, calculated based on band intensities, assessing PSAT1 alternative splicing events following HNRNPM knockdown (C) or overexpression (D) in CRC cells. Data are presented as mean ± SD of three independent experiments in C and D. Statistical analysis was performed by two-tailed unpaired Student's *t*-test (ns, *P* > 0.05).
